# Convergent death programs in chronic obstructive pulmonary disease: how pyroptotic and ferroptotic crosstalk reshapes therapeutic paradigms

**DOI:** 10.3389/fimmu.2026.1827181

**Published:** 2026-05-13

**Authors:** Aisi Huang, Shiming Liu, Jiao Lan

**Affiliations:** Shenzhen Baoan Traditional Chinese Medicine Hospital, Guangzhou University of Chinese Medicine, Shenzhen, Guangdong, China

**Keywords:** COPD, ferroptosis, inflammasome, inflammatory endotype, pyroptosis

## Abstract

Chronic obstructive pulmonary disease (COPD) remains a leading cause of global mortality, yet current therapies principally target bronchodilation and broad anti-inflammatory suppression rather than the regulated cell death programs driving tissue destruction. Pyroptosis, executed through inflammasome-driven gasdermin pore formation, and ferroptosis, mediated by iron-catalyzed lipid peroxidation upon GPX4 failure, have each been implicated in COPD pathogenesis but are conventionally treated as independent processes. This review advances three original contributions. First, we map pyroptotic and ferroptotic associations across COPD inflammatory endotypes, demonstrating preferential non-canonical pyroptotic engagement in T2-low neutrophilic disease and dual death-modality involvement in T2-high eosinophilic disease. Second, we delineate a hierarchical relationship—now supported in COPD-relevant epithelial systems—in which smoke-induced Nrf2 epigenetic silencing drives ferroptotic lipid peroxide accumulation that directly triggers pyroptotic execution through caspase-11 activation, positioning GPX4 as the molecular gatekeeper. Third, we propose an inverted U-shaped model reconciling paradoxical effects of lipid peroxidation on inflammasome regulation, where membrane phospholipid hydroperoxides drive activation while free cytosolic 4-hydroxynonenal mediates suppression through covalent NLRP3 modification. We further integrate extracellular trap biology as a convergent death-associated program sharing execution machinery with pyroptosis and ferroptosis. Building on this framework, we critically appraise emerging therapeutics—including dupilumab, anti-alarmin biologics, NLRP3 inhibitors, and ferroptosis-directed agents—and propose a biomarker-guided precision medicine strategy matching cell death-targeting therapies to individual inflammatory profiles. This framework reframes COPD therapeutic design from single-pathway inhibition toward integrated modulation of interconnected death programs stratified by inflammatory endotype.

## 1 Framing the problem: why two death modalities matter more than one

COPD remains the fourth leading cause of death globally, claiming roughly 3.5 million lives in 2023 (1), yet its pharmacological management continues to rely principally on bronchodilation and broad anti-inflammatory suppression rather than mechanistic intervention targeting the processes that drive tissue destruction (2). This disconnect between biological understanding and therapeutic capability demands re-examination of the pathological framework through which we interpret COPD.

The structural hallmarks of COPD—alveolar parenchymal destruction producing emphysema, and mucosal thickening within conducting airways—arise from a self-perpetuating inflammatory engine fueled primarily by tobacco smoke and secondarily by occupational or environmental particulates (3–5). Combustion products engage innate immune sensors including TLR2, TLR4, TLR9, and NLRP3, while simultaneously generating endogenous danger molecules that sustain immune cell mobilization long after the initial exposure (6, 7). The resulting oxidative burden and chronic signaling cascades constitute the accepted mechanistic foundation of progressive respiratory decline (8, 9).

However, this framework has historically treated COPD pathobiology as a relatively monolithic inflammatory process. What recent evidence demands we reconsider is that regulated cell death programs—specifically pyroptosis and ferroptosis—do not merely accompany this inflammation but actively orchestrate its perpetuation (10). Pyroptosis executes inflammatory cell lysis through inflammasome-driven gasdermin pore formation, coupling membrane rupture with substantial cytokine release (11). Ferroptosis destroys cells through iron-catalyzed lipid peroxidation that disrupts membrane integrity without engaging classical inflammatory caspases (12). The conventional assumption that these operate independently is now challenged by evidence of shared regulatory nodes spanning NF-κB, JAK-STAT, cGAS-STING, MAPK, and inflammasome signaling (10).

Complicating this picture is the recognition that COPD is not a unitary disease but a heterogeneous syndrome encompassing distinct inflammatory endotypes. Approximately 19–37% of patients exhibit elevated blood eosinophil counts (BEC ≥300 cells/µL) indicative of type 2 (T2) inflammation, characterized by increased airway expression of IL-4, IL-5, and IL-13 alongside basophil and mast cell involvement (13, 14). Crucially, this T2 signature is molecularly distinct from asthma: of genes associated with BEC in bronchial brush samples, only a single gene (CST1) overlapped between the two diseases (13, 15). The remaining patients display a T2-low phenotype dominated by neutrophilic inflammation and bacterial colonization (16, 17). However, these designations represent probabilistic, population-level tendencies rather than fixed biological states: sputum eosinophil counts show substantial within-patient variability on repeated sampling, with a meaningful proportion of patients shifting between “eosinophilic” and “non-eosinophilic” categories over time; bacterial colonisation occurs across eosinophil strata, albeit at different frequencies; and individual patients may simultaneously harbour features of both inflammatory profiles. GOLD guidance accordingly uses blood eosinophil counts primarily as a predictive biomarker for ICS responsiveness—with benefit minimal at <100 cells/µL and strongest at ≥300 cells/µL—rather than as a deterministic endotype label, emphasising that clinical context including exacerbation history and pneumonia risk remains essential in therapeutic decision-making.

This endotype spectrum raises a question that will recur throughout this review: do pyroptotic and ferroptotic death programs associate differentially with T2-high versus T2-low inflammatory profiles in COPD, and could mapping this relationship unlock more precise therapeutic targeting?

Previous reviews have examined pyroptosis and ferroptosis individually in COPD or catalogued their co-occurrence in respiratory diseases such as ARDS and acute lung injury (10, 18, 19). The present review advances beyond this prior work in three respects. First, we map pyroptotic and ferroptotic associations across COPD inflammatory profiles, demonstrating that T2-low, neutrophilic disease shows preferential engagement of non-canonical pyroptotic signaling linked to bacterial colonisation, while T2-high, eosinophilic disease more frequently involves both death modalities through alarmin-mediated and oxidative mechanisms—recognising these as probabilistic associations rather than deterministic assignments. Second, we identify a hierarchical relationship in which ferroptotic lipid peroxide accumulation, driven by smoke-induced Nrf2 epigenetic silencing, triggers pyroptotic execution through caspase-11 activation, with GPX4 functioning as the molecular gatekeeper. Third, we propose an inverted U-shaped model reconciling the paradoxical effects of lipid peroxidation intensity on inflammasome activation, with direct implications for therapeutic dose optimization.

**Figure 1:**
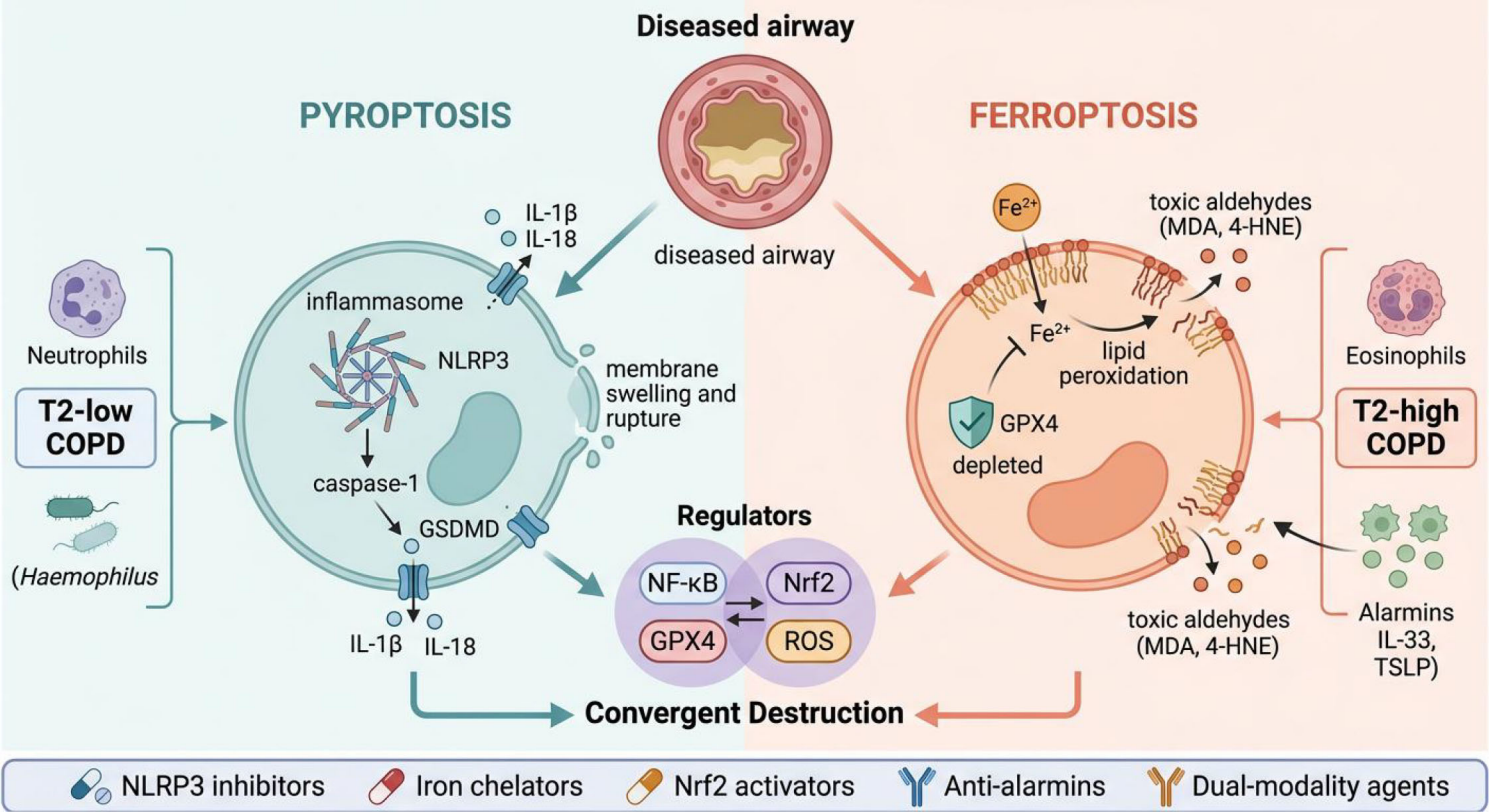
Convergent pyroptotic and ferroptotic death programs in COPD. Cigarette smoke simultaneously engages pyroptosis (left, teal) via inflammasome–caspase–GSDMD signaling and ferroptosis (right, coral) via iron-catalyzed lipid peroxidation upon GPX4 failure. Shared regulatory nodes (NF-κB, Nrf2, ROS) occupy the convergence zone (center, purple). T2-low COPD is primarily driven by non-canonical pyroptosis, while T2-high COPD engages both death modalities. Candidate therapeutic targets are indicated at bottom.

The central thesis advanced here is that treating pyroptosis and ferroptosis as mechanistically intertwined—rather than parallel—death programs reframes both our understanding of COPD pathogenesis and the design principles for next-generation therapies (**Figure 1**).

## 2 Pyroptotic machinery: from inflammasome assembly to membrane collapse

### 2.1 A death modality defined by inflammatory intent

Pyroptosis was first observed in *Salmonella*-infected macrophages and dendritic cells, though technological limitations initially caused its misclassification as apoptosis (20, 21) Subsequent work revealed that diverse bacterial pathogens—*Shigella* (22), *Listeria (*23), *Pseudomonas aeruginosa (*24), *Legionella pneumophila (*25) -trigger caspase-1-dependent destruction across multiple cell lineages (26, 27), and that endogenous danger signals equally initiate this death modality (28).

The defining morphological signature combines membrane permeabilization and dramatic cellular swelling with chromatin condensation and cytokine efflux, yet mitochondrial architecture remains intact and cytochrome C stays sequestered (29). This selective preservation is informative: pyroptosis preferentially targets plasma membrane function while sparing organellar compartments, suggesting that inflammatory payload delivery to the extracellular space—rather than intracellular metabolic collapse—represents its primary biological purpose. Understanding this inflammatory intent is essential for appreciating why pyroptosis is so destructive in COPD, where chronic activation transforms a host defense mechanism into a sustained tissue-damaging process.

### 2.2 Canonical and non-canonical activation: distinct triggers, converging destruction

The canonical pathway initiates when pattern recognition sensors—organized principally into AIM2, NLRC4, NLRP1, NLRP3, and PYRIN inflammasomes (30)—detect activating stimuli and assemble with ASC adaptor proteins and pro-caspase-1 (31). Mature caspase-1 then performs two concurrent proteolytic tasks: processing pro-IL-1β and pro-IL-18 into bioactive cytokines, and liberating GSDMD-N fragments that oligomerize within plasma membranes to construct pores permitting cytokine egress and ion influx (32, 33).

The non-canonical pathway bypasses the sensor–adaptor architecture of canonical inflammasome signaling entirely. Instead, gram-negative bacterial lipopolysaccharide (LPS) must gain access to the cytoplasm to directly engage caspase-4/5 (human) or caspase-11 (murine) via their N-terminal CARD domains (34). Cytoplasmic LPS delivery occurs through multiple mechanistically distinct routes that vary by biological context. Outer membrane vesicles (OMVs) constitutively shed by gram-negative bacteria—including the COPD-relevant pathogens Haemophilus influenzae and Moraxella catarrhalis—are internalised via endocytosis and deliver LPS to the cytosol through endosomal membrane fusion (34). During intracellular bacterial infection, LPS becomes accessible through pathogen escape from vacuolar compartments. Additionally, interferon-inducible guanylate-binding proteins (GBPs), particularly GBP1, GBP2, and GBP4, attack both free bacteria and pathogen-containing vacuole membranes, liberating LPS into the cytosol and serving as essential co-factors for efficient caspase-4/11 oligomerisation and activation (35, 36). A further route involves HMGB1-mediated extracellular LPS internalisation via the RAGE receptor followed by lysosomal destabilisation and cytosolic LPS release (37). In the COPD airway, OMV-mediated delivery from colonising gram-negative bacteria and GBP-dependent mechanisms in IFN-γ-primed alveolar macrophages are the most plausible—though not yet directly demonstrated—routes for sustained non-canonical inflammasome engagement. These caspases cleave GSDMD independently but cannot directly process IL-1β or IL-18 (38, 39). However, potassium efflux through the resulting GSDMD pores secondarily activates NLRP3 inflammasomes, indirectly enabling cytokine maturation (39). Caspase-11 additionally cleaves pannexin-1 channels to release ATP, engaging P2X7 receptors that establish potassium efflux conduits feeding back into NLRP3 assembly (40). Caspase-8 further integrates these circuits by facilitating ASC oligomerization and caspase-1 processing (41).

A distinction that will prove critical throughout this review concerns the relationship between inflammasome activation and pyroptotic cell death. These are mechanistically linked but experimentally dissociable events. Inflammasome activation—encompassing sensor oligomerisation, ASC speck formation, caspase-1 auto-processing, and maturation of IL-1β and IL-18—can occur without obligate progression to plasma membrane lysis. GSDMD cleavage generates the N-terminal pore-forming fragment (GSDMD-N), but pore formation at the plasma membrane is modulated by membrane lipid composition, cellular repair mechanisms (including ESCRT-III-dependent membrane shedding), and the subcellular trafficking of GSDMD-N itself. The most striking demonstration of this dissociation occurs in neutrophils, where GSDMD-N traffics predominantly to azurophilic granule membranes and autophagosomes rather than the plasma membrane, enabling IL-1β release through non-lytic secretory pathways without plasma membrane rupture (42). This means that detection of mature IL-1β or even cleaved GSDMD in a biological compartment does not necessarily indicate that pyroptotic cell death has occurred; it may instead reflect inflammasome-dependent cytokine processing and secretion from intact, viable cells. This distinction has direct implications for interpreting inflammatory mediator measurements in COPD sputum and BAL fluid, and we maintain it throughout the following sections.

**Figure 2:**
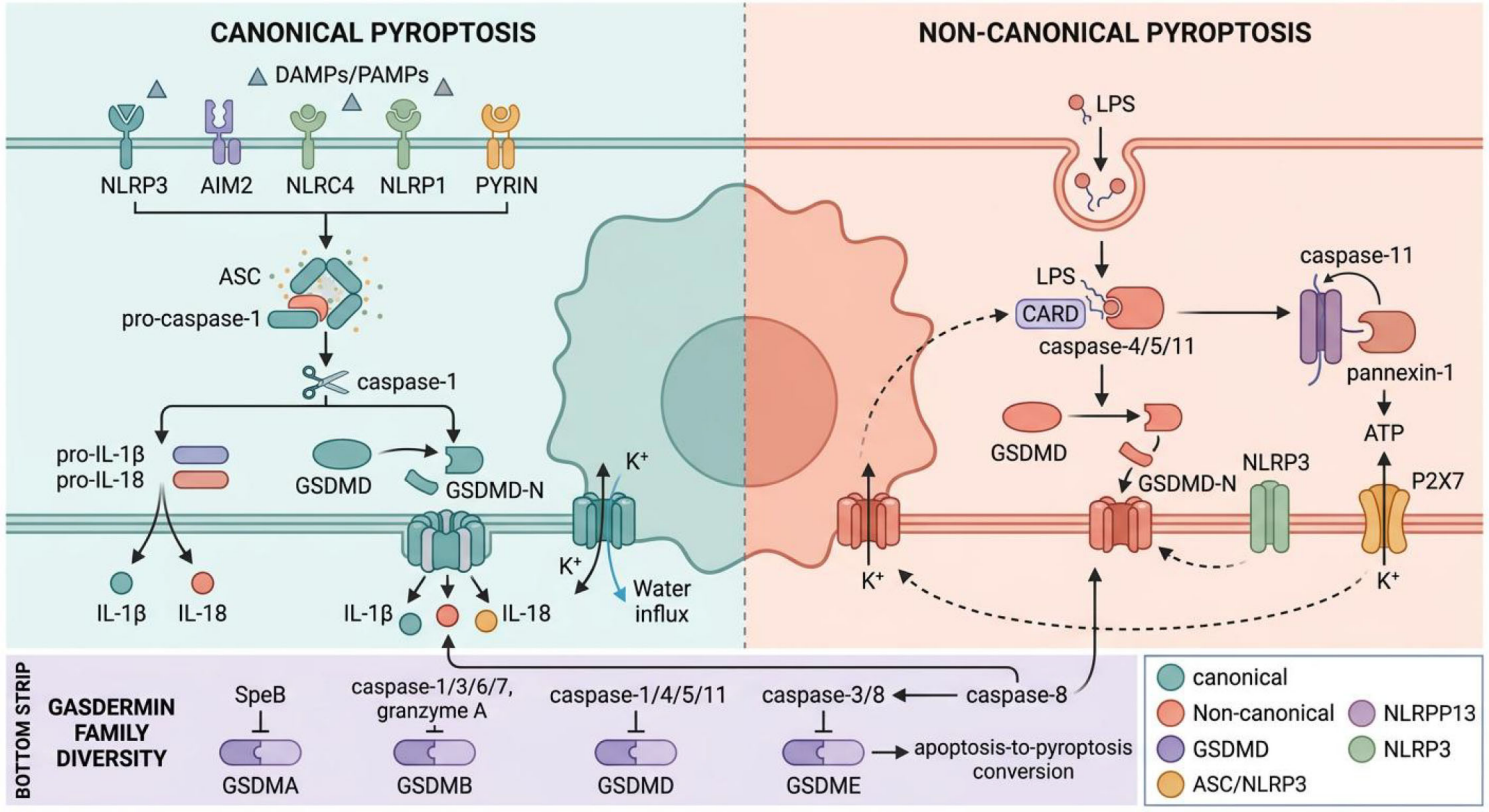
Canonical and non-canonical pyroptotic signaling pathways and gasdermin family diversity. The canonical pathway (left) proceeds through inflammasome assembly, caspase-1 activation, and GSDMD-N pore formation with IL-1β/IL-18 release. The non-canonical pathway (right) is triggered by cytoplasmic LPS engaging caspase-4/5/11, which cleaves GSDMD independently of caspase-1 and secondarily activates NLRP3 via K^+^ efflux. Caspase-8 integrates both circuits. The gasdermin family panel (bottom) shows GSDMA, GSDMB, GSDMD, and GSDME with their respective activating proteases.

This layered architecture raises a question directly relevant to COPD pathology: the T2-low endotype is characterized by bacterial colonization, particularly with gram-negative *Haemophilus influenzae (*43, 44), whose LPS would engage non-canonical pyroptotic signaling. By contrast, T2-high COPD, where bacterial load is paradoxically reduced (17, 45), may rely more heavily on canonical inflammasome activation through endogenous danger signals. If this endotype-specific activation pattern holds, it would imply that therapeutic targeting of specific caspases or inflammasome platforms should be guided by the patient’s inflammatory profile—an application of precision medicine principles to cell death biology (**Figure 2**).

### 2.3 Gasdermin diversity and therapeutic implications

The pyroptotic effector repertoire extends well beyond GSDMD. Caspase-3 cleaves GSDME during apoptotic progression, enabling death modality conversion (46, 47). Caspase-8 processes both GSDMD and GSDME while activating NLRP3 inflammasomes (48–50). Streptococcal SpeB protease cleaves GSDMA during bacterial colonization (51), and GSDMB undergoes processing by caspase-1/3/6/7 and granzyme A while simultaneously augmenting caspase-4-mediated GSDMD cleavage (52).

This gasdermin diversity has a direct therapeutic consequence that deserves emphasis: strategies targeting a single gasdermin or caspase will likely prove insufficient, since compensatory pathways through alternative family members could maintain inflammatory output. Effective pyroptosis-directed therapy will probably require targeting upstream converging signals—such as the oxidative triggers or NF-κB priming events—rather than individual executioners. This insight becomes particularly relevant when we consider the convergence with ferroptosis discussed in Section 8, where shared upstream regulators may offer intervention points affecting both death modalities simultaneously.

## 3 Ferroptotic death: iron, lipids, and the failure of membrane defenses

### 3.1 Biochemical architecture of a non-inflammatory death

Ferroptosis, formally named in 2012 though observed earlier in RAS-mutant cells (53), is defined by iron-dependent lipid peroxidation that destroys membrane integrity without engaging classical inflammatory caspases (54). Excess intracellular iron fuels Fenton chemistry to generate reactive oxygen species that attack membrane-embedded polyunsaturated fatty acids, producing toxic aldehydes—MDA, PLOOH, and 4-HNE (55). Two enzymatic steps prepare the susceptible substrate: ACSL4 conjugates coenzyme A to PUFA chains, and LPCAT3 esterifies these products into membrane phospholipids (56, 57). Multiple inducer classes exploit different defensive vulnerabilities: erastin depletes glutathione by blocking system Xc^-^ while promoting GPX4 degradation; RSL3 and DPI7 directly inactivate GPX4; FIN56 degrades GPX4 while depleting CoQ10; and FINO2 oxidizes labile iron directly (58, 59).

### 3.2 Defensive architecture and its failure in disease

GPX4 functions as the principal enzymatic shield, utilizing glutathione to reduce lipid hydroperoxides within membranes (60, 61). The transcription factor Nrf2 governs this defense hierarchy, driving expression of GPX4, HO-1, and other antioxidant genes while regulating SLC7A11 through Keap1 signaling (62–66). When this architecture fails—whether through glutathione depletion, GPX4 inactivation, or Nrf2 suppression—membrane peroxidation proceeds unchecked toward lethal thresholds.

**Figure 3:**
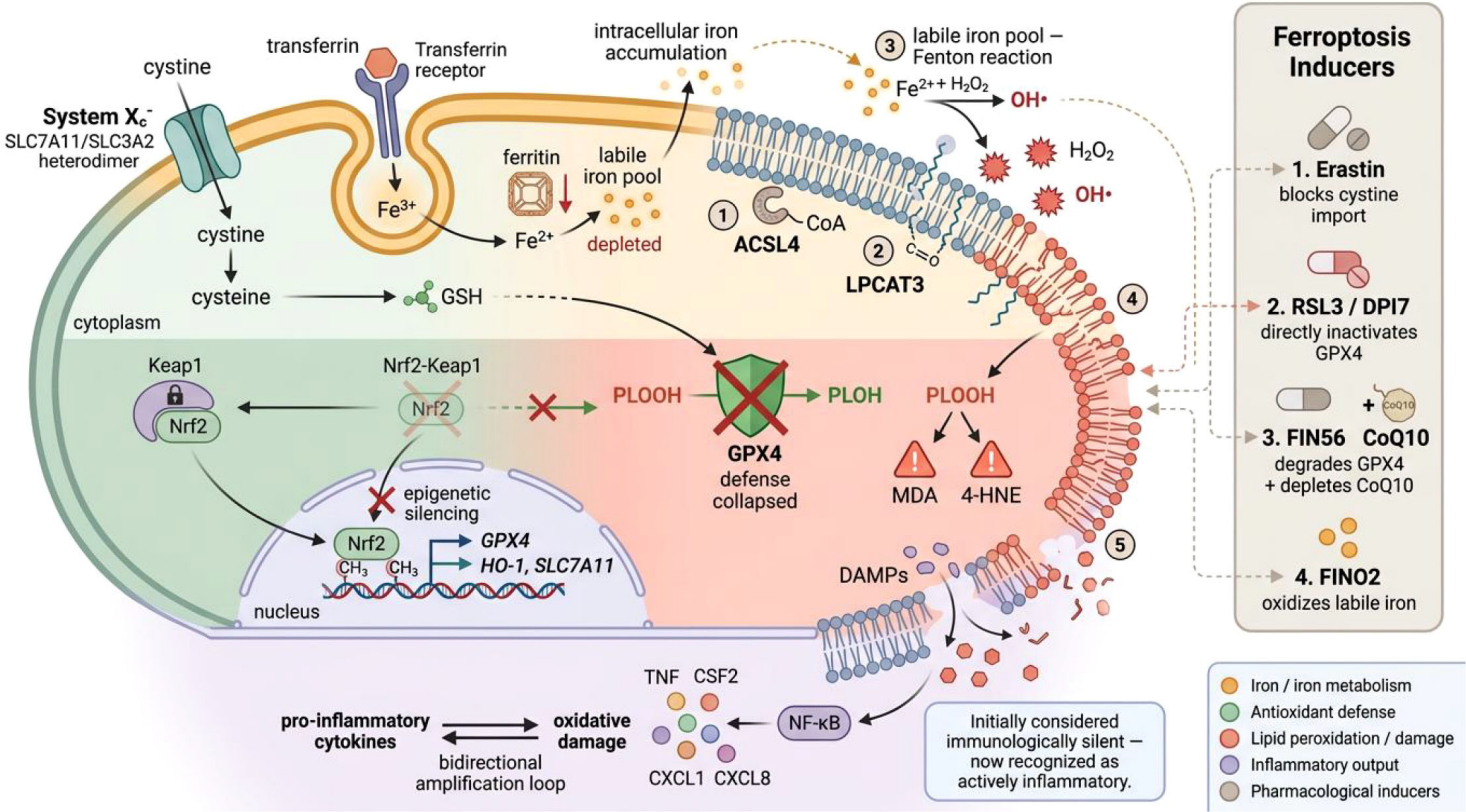
Ferroptosis signaling mechanism: iron dysregulation, lipid peroxidation, defense failure, and inflammatory output. Iron enters via TfR1 and accumulates as Fe²^+^ in the labile iron pool; ferritin storage is depleted. System Xc^-^ imports cystine for GSH synthesis, which sustains GPX4 activity at the membrane. Nrf2 epigenetic silencing (red X) and GPX4 depletion remove antioxidant defenses. ACSL4 and LPCAT3 prepare PUFA-containing membrane substrates; Fe²^+^-driven Fenton chemistry generates hydroxyl radicals that initiate lipid peroxidation, producing MDA and 4-HNE and culminating in membrane rupture. Released DAMPs activate NF-κB-dependent cytokine secretion with bidirectional amplification. Right panel: four inducer classes and their molecular targets.

A conceptual point that will become critical later: ferroptosis was initially characterized as immunologically silent, a distinction from pyroptosis’s overtly inflammatory nature. However, accumulating evidence reveals that ferroptotic cell death actively generates inflammatory signals. Lipid peroxidation products activate NF-κB in dying smooth muscle cells, driving secretion of TNF, CSF2, CXCL1, and CXCL8 (67), while pharmacological lipid peroxidation inhibition suppresses TNF-α, IL-1β, and IL-6 in hepatic injury models (68). Pro-inflammatory cytokines reciprocally intensify oxidative damage, creating bidirectional amplification (69, 70). This inflammatory dimension is what enables ferroptosis to participate in crosstalk with pyroptosis—a connection that forms the mechanistic core of this review (**Figure 3**).

## 4 Pyroptosis in COPD: a multi-layered assault on airway integrity

### 4.1 Inflammasome activation as the central pathological event

NLRP3 inflammasome engagement is a consistently observed feature of COPD pathobiology that has been documented across multiple cellular compartments, though the evidence varies in strength and mechanistic detail between compartments.

#### 4.1.1 Bronchial and alveolar epithelium

In bronchial epithelial cells, cigarette smoke extract (CSE) activates NLRP3 protein expression and complex assembly, with downstream caspase-1 activation leading to GSDMD cleavage and measurable compromise of epithelial barrier integrity *in vitro (*71, 72). The upstream trigger cascade in this compartment is multilayered: CSE-generated reactive oxygen species provoke endoplasmic reticulum stress engaging PERK/eIF2α/CHOP signaling, which elevates TXNIP concentrations that potentiate NLRP3 activation (52). Zhang et al. demonstrated in 16HBE cells (a human bronchial epithelial cell line) that CSE induced the full pyroptotic morphological sequence—GSDMD-N membrane translocation, propidium iodide uptake indicating membrane permeabilisation, and IL-1β release—through the ROS/NLRP3/caspase-1 axis, and that ROS scavenging suppressed this cascade more effectively than targeting individual downstream components (72). These findings were obtained in immortalised cell lines exposed to acute CSE concentrations; whether the same complete pyroptotic sequence occurs in primary differentiated airway epithelial cells under chronic, lower-level smoke exposure remains less well characterised. Clinical specimens from COPD patients show elevated NLRP3 mRNA and protein in bronchial biopsies compared with non-COPD controls (73), but these cross-sectional observations do not distinguish between cells that have undergone priming (NLRP3 upregulation without activation) and those exhibiting active inflammasome assembly. Hydrogen sulfide donors suppress TLR4-dependent NLRP3 priming in bronchial epithelial models (74), and epicatechin similarly attenuates CSE-induced epithelial inflammation by suppressing ROS-linked NLRP3 activation (75), identifying the oxidative priming event as a potentially efficient intervention point in this compartment.

#### 4.1.2 Alveolar macrophages and myeloid cells

The macrophage compartment provides the strongest evidence for functional inflammasome activation in COPD. Alveolar macrophages from COPD patients release significantly more IL-1β upon ex vivo LPS stimulation than those from matched smokers without airflow limitation, indicating a primed inflammasome state (73). TREM-1 expression in alveolar macrophages drives pulmonary injury through NLRP3-dependent pathways in murine COPD models, and TREM-1 blockade reduces both structural damage and inflammasome activation markers including caspase-1 cleavage and IL-1β release (76). The p38 MAPK pathway independently governs macrophage inflammasome output: the p38 inhibitor doramapimod suppressed TNF-α and IL-6 from COPD patient alveolar macrophages more effectively than budesonide in ex vivo assays (77, 78), a finding with direct clinical implications given the known corticosteroid resistance in subpopulations of COPD patients. TLR4/MyD88/NF-κB signaling provides the canonical priming signal for NLRP3 in this compartment (79–81), and smoke-induced emphysema models confirm TLR4 involvement in fibrosis, emphysema, and pulmonary function deterioration (82, 83). Whether macrophage inflammasome activation in these models proceeds to full pyroptotic lysis—as opposed to sublytic GSDMD pore formation with cytokine release and subsequent membrane repair—has not been systematically assessed with live-cell imaging in COPD-specific systems, though the lytic endpoint is typically assumed based on caspase-1 and LDH release measurements.

#### 4.1.3 Neutrophils: inflammasome activation without obligate pyroptotic death

The neutrophil compartment presents a fundamentally different relationship between inflammasome activation and cell fate, and this relationship is more nuanced than previously framed. As introduced in Section 2.2, GSDMD-N in neutrophils traffics predominantly to intracellular organelle membranes — azurophilic granules and autophagosomes — rather than to the plasma membrane, enabling mature IL-1β secretion through non-lytic pathways without plasma membrane rupture. However, this non-lytic secretory fate is not the only consequence of neutrophil inflammasome activation. Accumulating evidence establishes that inflammasome signaling and extracellular trap formation are mechanistically interdependent rather than parallel programs. Chen et al. demonstrated that non-canonical caspase-11/GSDMD signaling in neutrophils directly elicits NET extrusion, with GSDMD pore formation serving as a shared execution node for both pyroptotic membrane rupture and NET release (84). Münzer et al. subsequently showed that NLRP3 inflammasome activation in neutrophils licenses a specific NET release program through a GSDMD-dependent mechanism distinct from classical “suicidal” NETosis (85). Together, these findings establish that inflammasome-activated neutrophils in COPD can adopt at least three distinct fates: (i) non-lytic IL-1β/IL-18 secretion with preserved viability; (ii) NET extrusion driven by GSDMD-dependent mechanisms without obligate progression to full pyroptotic lysis; or (iii) lytic pyroptotic death with membrane rupture. The balance between these fates likely depends on the intensity of inflammasome activation, the subcellular distribution of GSDMD-N, and local oxidative and metabolic context.

This revised understanding has profound implications for interpreting COPD sputum data and for therapeutic targeting. The elevated IL-1β and IL-18 concentrations consistently measured in COPD sputum (86, 87) may reflect any combination of non-lytic secretion, lytic pyroptotic death, and NET-associated cytokine release; the canonical assumption that these markers indicate pyroptotic cell death — and that neutrophils functioning as sustained cytokine sources are simply “pyroptosis-resistant” — is incomplete. More accurately, inflammasome-activated neutrophils in the COPD airway likely distribute across a spectrum of outcomes, with NET extrusion representing a major pathological output in its own right. This reframing directly informs Section 7.4 (convergence with ferroptosis) and modifies the therapeutic rationale in Section 10.2: interventions targeting GSDMD would be expected to attenuate both pyroptotic lysis and GSDMD-dependent NETosis simultaneously, whereas NLRP3-directed therapies may reduce cytokine output and NET release without necessarily affecting the non-lytic, GSDMD-independent secretory pathway.

### 4.2 Epithelial alarmins: linking pyroptotic cell death to inflammatory endotype determination

Chronic smoke exposure releases epithelial alarmins—TSLP and IL-33—that coordinate both T2 and non-T2 inflammatory responses (88). The relationship between pyroptotic cell death and IL-33 signaling is more nuanced than a simple “pyroptosis → alarmin release” model, requiring consideration of three regulatory layers.

First, regarding release and proteolytic processing: full-length IL-33 is bioactive upon release and does not require inflammasome processing. Critically, caspase-1 cleaves IL-33 within its cytokine domain, inactivating rather than maturing the protein (89). IL-33 release therefore occurs predominantly through non–caspase-mediated necrotic lysis (including GSDMD-dependent membrane rupture before caspase-1 access) and active secretion from mechanically stressed epithelial cells. Once extracellular, neutrophil serine proteases—elastase, cathepsin G, and proteinase 3—generate mature 18–21 kDa forms with 10- to 30-fold greater ST2 activity (88), aligning IL-33 maturation with the neutrophilic T2-low endotype.

Second, regarding redox-dependent signaling: reduced IL-33 (IL-33^red^) rapidly oxidises extracellularly to IL-33^ox^ through intramolecular disulfide bond formation. IL-33^ox^ signals through RAGE/EGFR rather than ST2, driving epithelial damage and neutrophil recruitment (90). Tozorakimab, which binds both forms, is designed to neutralise both modalities. This redox switch connects directly to our convergence framework: the same oxidative environment driving ferroptotic PLOOH accumulation accelerates the IL-33^red^ → IL-33^ox^ transition. Environments with preserved Nrf2/GSH/GPX4 tone favour IL-33^red^–ST2–ILC2 signaling (T2-high), whereas advanced ferroptotic burden preferentially generates IL-33^ox^ and shifts toward RAGE/EGFR-associated neutrophilic pathology (T2-low).

Third, IL-33^red^ directly drives eosinophil extracellular trap (EET) release through an ST2–IL-5–mitochondrial ROS axis (91), creating a causal chain from alarmin release to EETosis in T2-high environments—mechanistically distinct from neutrophilic NET release in T2-low disease.

IL-33 protein levels and IL-33-positive small airway epithelial cells are elevated in COPD (92, 93), with expression concentrated in basal cells and increased in remodeled airways (94, 95). TSLP is similarly increased in COPD biopsies and sputum (96, 97), bridging innate danger sensing to adaptive immune polarization through dendritic cell and ILC2 activation (98, 99).

Integrating these layers: canonical caspase-1 pyroptosis acts as a brake on IL-33 bioactivity (89), while GSDMD-dependent lysis and necrosis release active IL-33 ^red^; the ferroptosis-governed extracellular redox state determines whether IL-33 signals through ST2 or RAGE/EGFR (90). This predicts that ferroptosis inhibitors should modulate not only pyroptotic execution but also alarmin signaling mode—an untested but experimentally tractable prediction that provides rationale for combining anti-alarmin biologics with death-program-targeted therapies.

### 4.3 The microbiome dimension: correlation, mechanism, and the limits of current evidence

The interaction between pyroptosis and the pulmonary microbiome has been described with increasing frequency in the COPD literature, but the nature of this interaction — whether causal, bidirectional, or merely coincident — warrants more careful parsing than it has typically received.

The descriptive data are clear. Up to 76.7% of COPD patients harbour increased abundances of potentially pathogenic bacteria in the stable state, with reduced diversity and overrepresentation of Proteobacteria including Haemophilus influenzae and Moraxella catarrhalis (100, 101). Cross-sectional analyses show that sputum neutrophil counts correlate positively with bacterial load, while eosinophil counts show an inverse relationship (16, 17, 45). ICS use, which preferentially benefits eosinophilic patients, has been associated with reduced airway bacterial diversity and increased pathogenic species (44, 102, 103). These findings are reproducible and clinically important, but they are associative.

A genuinely mechanistic claim — that bacterial colonisation drives pyroptosis in COPD airways — requires stronger evidence than the existing human data provide. What is mechanistically established, in experimental systems, is a biochemical pathway rather than a clinical one: gram-negative bacterial LPS delivered into the cytoplasm (including via outer membrane vesicles) engages caspase-4/5/11 to cleave GSDMD, and H. influenzae components can trigger inflammasome activation in cultured macrophages and bronchial epithelial cells (34, 38, 39). Extrapolating from these systems to the chronically colonised human airway, however, requires several assumptions that have not been independently verified: that intracellular LPS delivery occurs at pathologically relevant concentrations *in vivo*; that caspase-4/5/11 activation is sustained rather than transient; and that pyroptotic output in colonised patients exceeds what would be produced by smoke exposure alone.

At least three alternative interpretations remain compatible with available data: (i) a shared upstream driver—smoke-induced epithelial barrier damage—could independently promote both bacterial persistence and pyroptotic priming without direct causation; (ii) pyroptosis-mediated barrier disruption may precede and facilitate bacterial colonisation (72, 104); and (iii) neutrophilic inflammation itself may drive pyroptotic activation through ROS-mediated NLRP3 priming, with bacteria serving as markers of a permissive milieu rather than initiating stimuli.

Resolving these possibilities will require study designs that the field has not yet delivered. Longitudinal cohorts tracking microbiome composition, inflammasome biomarkers (IL-1β, IL-18, GSDMD fragments; see Section 5.4), and epithelial barrier integrity within the same patients over time could establish temporal precedence. Interventional studies — targeted antibiotic regimens, phage therapy, or experimental medicine designs using NLRP3 inhibitors in colonised versus non-colonised patients — could test directionality directly. Ideally, genetic instruments influencing innate immune tone (for example, NLRP3 or CARD8 variants) could be used in Mendelian-randomisation frameworks to probe causality, although the relatively modest effect sizes of such variants make this technically demanding.

**Figure 4:**
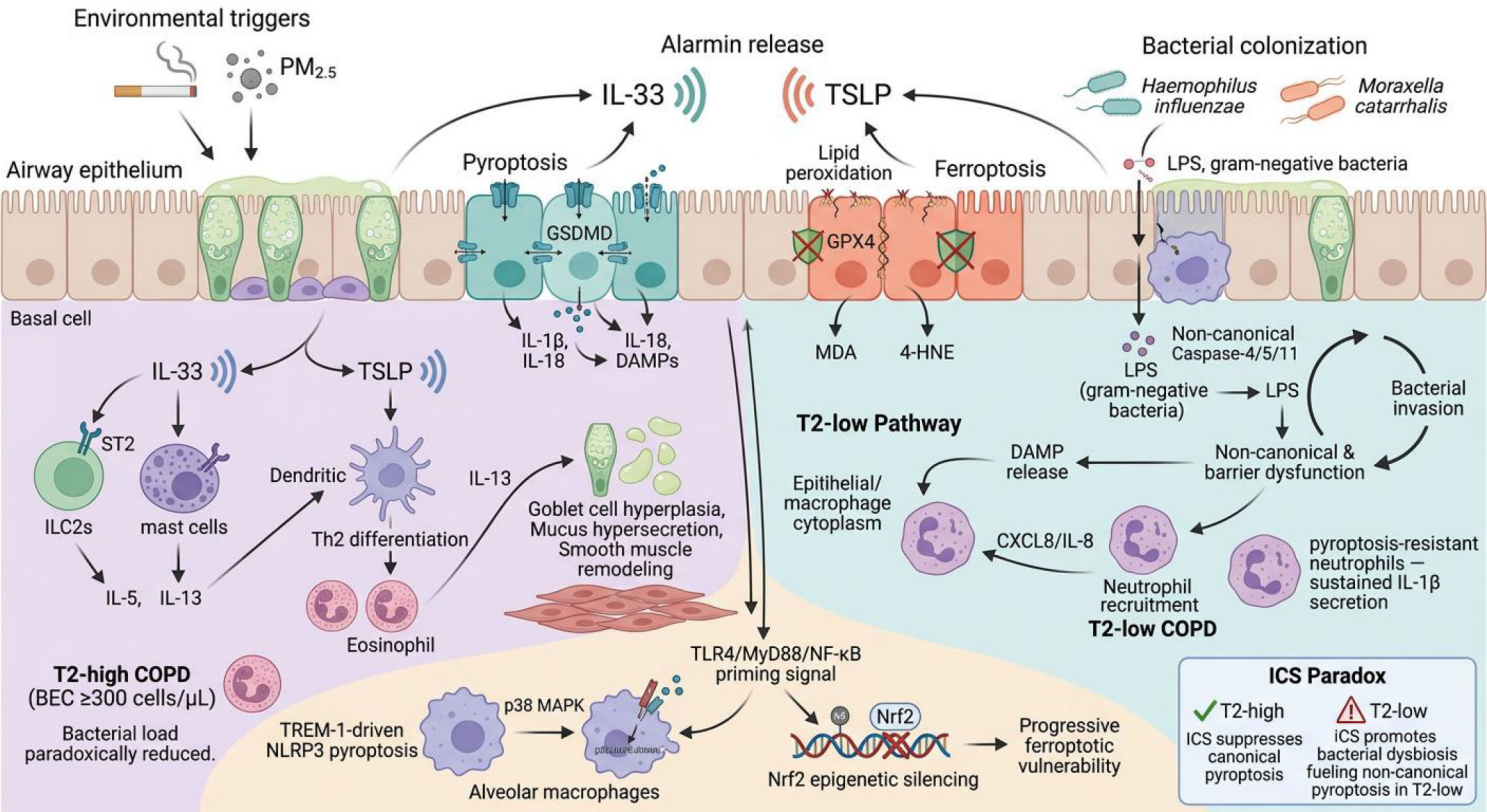
Integrated pathological landscape of pyroptosis and ferroptosis in the COPD airway. Cigarette smoke triggers concurrent pyroptotic (teal) and ferroptotic (coral) epithelial death. The T2-high pathway (left, lavender) proceeds through IL-33/TSLP–ILC2 activation, IL-5/IL-13 production, and goblet cell hyperplasia. The T2-low pathway (right) involves gram-negative bacterial LPS-driven non-canonical pyroptosis and persistent neutrophilic inflammation. Nrf2 epigenetic silencing creates progressive ferroptotic vulnerability in macrophages. The ICS paradox inset illustrates how corticosteroids suppress canonical pyroptosis in T2-high patients while promoting bacterial dysbiosis that fuels non-canonical pyroptosis in T2-low patients.

The therapeutic implications flagged in the original framing nonetheless retain value, provided they are stated as hypotheses rather than established mechanisms. The clinical observation that ICS both suppresses eosinophilic inflammation and alters microbiome composition generates a testable prediction: cell death pathway–specific therapeutics (for example, selective NLRP3 or caspase-4/11 inhibitors) may produce anti-inflammatory benefit without the dysbiosis associated with broad corticosteroid immunosuppression. The preliminary finding that benralizumab-mediated eosinophil depletion did not significantly alter airway microbiome composition (105) offers partial reassurance that T2-targeted biologics may avoid exacerbating dysbiosis, but equivalent data for pyroptosis-directed interventions are absent. Until such data exist, the “vicious cycle” model — bacterial colonisation drives pyroptosis, which disrupts barriers, which permits further colonisation — should be treated as a heuristic that organises existing observations and motivates future experiments, rather than as a demonstrated mechanism (**Figure 4**).

## 5 Ferroptosis compounds COPD tissue destruction through parallel but interconnected mechanisms

### 5.1 The ferroptotic signature in smoke-exposed airways

Murine lung epithelial cells exposed to cigarette smoke accumulate excess iron and undergo enhanced lipid peroxidation, triggering non-apoptotic death that GPX4 normally restrains. Both deferoxamine and ferrostatin-1 attenuate this damage, identifying iron chelation and lipid radical trapping as viable therapeutic strategies (106). The ferroptotic signature manifests consistently across experimental systems: smoke exposure elevates MDA concentrations and iron content while suppressing GPX4, depleting ferritin, and upregulating transferrin receptor—collectively indicating disrupted iron homeostasis converging on lethal lipid peroxidation (107). Environmental particulate matter (PM2.5) produces parallel changes by increasing endothelial iron and ROS while depleting glutathione and NADPH (108).

### 5.2 Epigenetic vulnerability and progressive ferroptotic susceptibility

A particularly consequential finding is that Nrf2—the master transcriptional regulator of antioxidant defense—undergoes epigenetic silencing through CpG hypermethylation at its promoter in COPD lung tissue (109). This creates a self-reinforcing destructive cycle: initial oxidative injury silences the very gene responsible for mounting an antioxidant response, rendering tissue progressively more vulnerable to subsequent ferroptotic stimuli. Restoring Nrf2 function elevates GPX4 and inhibits bronchial epithelial ferroptosis (109), validating this epigenetic modification as a therapeutic target. Complementary protective mechanisms include dihydroquercetin-mediated Nrf2 activation to increase SLC7A11 and GPX4 (110, 111), curcumin reversal of smoke-induced iron dysregulation (107), and hydrogen sulfide restoration of redox balance through the Nrf2-PPAR-ferritin pathway (111). Exosomal miR-26a-5p from endothelial progenitor cells offers an additional protective axis by mitigating smoke-induced ferroptosis through the PTGS2/PGE2 pathway (112).

An important translational question is whether smoke-induced Nrf2 promoter hypermethylation is reversible. Emerging evidence suggests that it is, at least partially. DNA methyltransferase inhibitors such as 5-azacytidine and decitabine have been shown to restore Nrf2 expression in hypermethylated cancer cell lines, and the dietary methyl donor S-adenosylmethionine modulates global methylation patterns in oxidatively stressed tissues. More targeted approaches include ten-eleven translocation (TET) enzyme activation, which catalyses oxidative demethylation of 5-methylcytosine, and CRISPR-dCas9-TET1 fusion constructs that enable locus-specific demethylation of the Nrf2 promoter—though these remain at the proof-of-concept stage. Among clinically available agents, sulforaphane and dimethyl fumarate activate Nrf2 through Keap1 modification rather than epigenetic reversal, but may partially compensate for reduced Nrf2 transcription by stabilising the existing protein pool. Whether combining epigenetic reactivation strategies with Keap1-dependent Nrf2 stabilisers could achieve sufficient GPX4 restoration to interrupt the ferroptosis–pyroptosis convergence described in Section 8 remains untested but represents a logical therapeutic priority.

### 5.3 Ferroptosis, corticosteroid resistance, and the smoking paradox: evidence, hypothesis, and limitations

A connection warranting careful consideration links ferroptotic vulnerability to the reduced efficacy of inhaled corticosteroids (ICS) among current smokers, though the supporting evidence is predominantly indirect and the mechanistic chain must be stated with appropriate caution.

What is directly established. Multiple clinical analyses have reported diminished ICS clinical effect in active smokers (113, 114), with oxidative stress and innate immune dysregulation proposed as explanatory mechanisms (115). The best-characterised molecular bridge between the oxidative burden of smoking and blunted corticosteroid response is oxidative/nitrosative inactivation of histone deacetylase 2 (HDAC2), a cofactor required for glucocorticoid receptor-mediated transrepression of inflammatory genes. Reduced HDAC2 activity and expression have been repeatedly demonstrated in peripheral lung tissue and alveolar macrophages from COPD patients and smokers, and peroxynitrite-mediated tyrosine nitration, PI3Kδ activation, and glutathione depletion each contribute to this inactivation. Experimental antioxidant or PI3Kδ inhibitor intervention can partially restore steroid responsiveness in smoke-exposed macrophages. This HDAC2-oxidative axis therefore constitutes an established — though still indirect — mechanistic link between smoking-induced oxidative stress and corticosteroid insensitivity.

Where ferroptosis enters — and where it has not yet been directly tested. Ferroptotic biology shares substantial mechanistic territory with the HDAC2-oxidative inactivation axis: GPX4 depletion, glutathione consumption downstream of system Xc^-^ dysregulation, and Nrf2 epigenetic silencing (109) all expand the pool of reactive lipid species and peroxynitrite available to inactivate HDAC2. What is directly demonstrated in COPD-relevant systems is limited to: (i) reduced GPX4 protein in COPD lung tissue and smoke-exposed epithelium (106); (ii) Nrf2 promoter hypermethylation driving GPX4 suppression; and (iii) rescue of epithelial ferroptosis by deferoxamine, ferrostatin-1, and Nrf2 activators (106). What has not been directly demonstrated is that (a) ferroptotic lipid peroxidation causally reduces HDAC2 activity or corticosteroid responsiveness in COPD airway cells, (b) ferroptosis inhibitors restore ICS efficacy in smoke-exposed models, or (c) GPX4 manipulation alters steroid sensitivity independently of exogenous oxidative challenge. The causal arrow from ferroptosis to corticosteroid resistance, in other words, remains inferred rather than observed.

A hypothesis stated explicitly as such. We therefore propose — as a testable hypothesis rather than an established mechanism — that ferroptotic lipid peroxidation contributes to the oxidative burden underlying HDAC2 inactivation and thereby to ICS refractoriness in current smokers. This framing generates three specific, falsifiable predictions. First, in cigarette smoke extract-exposed primary human bronchial epithelial cells and alveolar macrophages, co-administration of ferrostatin-1 or deferoxamine should augment the anti-inflammatory effect of dexamethasone or budesonide on IL-8, TNF-α, and IL-6 output, with concomitant preservation of HDAC2 activity. Second, GPX4 overexpression should phenocopy ferroptosis inhibition in restoring steroid sensitivity, whereas conditional GPX4 knockdown should induce or intensify corticosteroid resistance independently of exogenous ROS. Third, in chronic smoke-exposure murine models, combined ICS plus ferrostatin-1 (or Nrf2 reactivation via promoter demethylation) should yield greater attenuation of neutrophilic inflammation, HDAC2 loss, and alveolar destruction than either agent alone. To our knowledge, none of these experiments has been reported in COPD-specific systems.

Several caveats warrant acknowledgement. The link between oxidative stress and corticosteroid resistance through HDAC2 biology is not specific to ferroptosis; mitochondrial dysfunction, NADPH oxidase, and iNOS-derived nitrosative stress contribute to the same oxidative pool. The clinical heterogeneity of smokers who respond poorly to ICS means that the proportion driven predominantly by ferroptotic mechanisms is unknown. Broad antioxidant trials (N-acetylcysteine, vitamin E) have been largely disappointing, though these employed non-selective agents and were not stratified by oxidative biomarkers, leaving the specific question of targeted ferroptotic inhibition genuinely untested. The inverse relationship — whether ICS themselves modulate ferroptotic markers in COPD airways — has similarly not been examined, despite its direct relevance to interpreting any future combination trial.

Supporting clinical observation. Within these limits, analyses of the FLAME and ISOLDE studies add an empirical anchor for the broader precision-medicine framework. BEC change during ICS treatment carries biomarker potential: in approximately 40% of patients BEC decreased on ICS, and these individuals showed greater clinical benefit; however, in 20% BEC paradoxically increased, accompanied by accelerated lung function decline and increased pneumonia risk (116, 117). This differential response is consistent with — though does not prove — heterogeneity in underlying cell death programs: patients whose inflammation is driven chiefly by pyroptosis-mediated alarmin release may respond to ICS-mediated inflammasome suppression, whereas those with predominant ferroptosis-driven oxidative pathology may resist corticosteroids and potentially require adjunctive antioxidant or ferroptosis-targeted strategies (**Figure 4**).

### 5.4 Clinical biomarker evidence in COPD patients

The mechanistic findings discussed above would remain largely academic unless they translate into measurable signatures in patients. Fortunately, accumulating clinical data now support the relevance of several ferroptosis-associated biomarkers in COPD, offering at least a preliminary foundation for translating pathway biology into clinical stratification.

Malondialdehyde (MDA), the terminal aldehyde product of polyunsaturated fatty acid peroxidation, is consistently elevated in COPD patients. Plasma and serum MDA concentrations are significantly higher in stable COPD compared with healthy smokers and non-smoking controls, and rise further during acute exacerbations (118, 119). MDA levels correlate inversely with FEV1% predicted and track with GOLD severity stages, and exhaled breath condensate MDA similarly discriminates COPD patients from controls and increases with symptom burden (120). These observations indicate that MDA captures, at least in part, the systemic oxidative burden that drives both ferroptotic and pyroptotic cascades described in the preceding sections.

4-Hydroxynonenal (4-HNE) protein adducts, a more stable and cell-localisable marker of lipid peroxidation, have been demonstrated by immunohistochemistry to be markedly increased in airway epithelium, alveolar epithelial cells, smooth muscle, and endothelium of COPD lung specimens compared with smokers without airflow limitation, with 4-HNE adduct burden correlating inversely with FEV_1_ (120). The cellular distribution—concentrated in epithelial and macrophage populations—maps directly onto the compartments implicated in pyroptotic–ferroptotic crosstalk (Section 8), reinforcing the mechanistic plausibility of convergence in human tissue rather than merely in model systems.

GPX4 downregulation has been documented at both transcriptomic and protein levels in COPD lungs. Yoshida et al. reported reduced GPX4 protein expression in lung tissue from COPD patients alongside corresponding murine smoke-exposure data (106), and Zhang et al. subsequently showed that Nrf2 promoter hypermethylation in COPD specimens drives downstream GPX4 suppression (109), mechanistically linking the epigenetic vulnerability described in Section 5.2 to the measurable protein deficit. Complementary iron homeostasis parameters reinforce this picture: serum and sputum ferritin levels are elevated in COPD, transferrin receptor expression is increased in airway epithelium, and bronchoalveolar lavage iron content has been reported to correlate with disease severity (120).

On the pyroptotic side, circulating and sputum IL-1β and IL-18 concentrations are consistently elevated in COPD and rise further during exacerbations, correlating with sputum neutrophil counts and bacterial load (86, 87). More recent proteomic and immunoassay work has begun to detect GSDMD cleavage fragments in COPD sputum and serum, offering a more specific indicator of pyroptotic execution than cytokine levels alone, although standardised assays are still lacking.

Taken together, these data support the feasibility of a biomarker panel combining MDA, 4-HNE, GPX4 (or its regulatory axis, e.g. Nrf2 methylation status), iron homeostasis parameters, and inflammasome output markers (IL-1β, IL-18, GSDMD fragments) for stratifying patients along the pyroptotic–ferroptotic axis. Several important gaps remain. Most published studies examined single markers in cross-sectional cohorts, and longitudinal data relating biomarker trajectories to exacerbation frequency, lung function decline, or treatment response are scarce. In addition, direct co-localisation studies of NLRP3, ACSL4, and GPX4 in human COPD airway tissue—analogous to those already performed in coronary atherosclerosis (121)—have not been reported, and would provide a critical test of whether the convergence biology operates in the diseased human lung *in situ* rather than solely in model systems.

## 6 Comparative insights from asthma: shared mechanisms, divergent contexts

While this review focuses on COPD, the parallel investigation of pyroptosis and ferroptosis in asthma provides comparative insights that illuminate both shared biology and disease-specific distinctions.

### 6.1 Ferroptosis in asthma: eosinophils as both targets and therapeutic vulnerabilities

Elevated airway iron in asthma triggers Fenton reaction-mediated epithelial ferroptosis, with resultant ROS activating pro-inflammatory cytokines that promote T2 inflammation (122). The 15LOX1-PEBP1 complex sustains IL-13/IL-4-mediated Th2 inflammation while simultaneously promoting ferroptosis (123), creating a mechanism where the same molecular machinery drives both inflammatory signaling and oxidative cell death. This dual function has a provocative therapeutic corollary: erastin, artesunate, or RSL3 combined with dexamethasone can selectively induce eosinophil ferroptosis, reducing airway inflammation while potentially enabling glucocorticoid dose reduction (42). Blocking the p38 MAPK pathway with Rhizoma Dioscoreae Nipponicae similarly inhibits asthma progression while upregulating eosinophil ferroptosis (124).

The selectivity challenge is obvious but critical: inducing ferroptosis in disease-perpetuating eosinophils while protecting structural epithelial and endothelial cells demands targeting precision that current pharmacology cannot reliably achieve. Nevertheless, this concept connects directly to COPD: if ferroptosis induction could selectively target activated inflammatory cells in T2-high COPD (where eosinophil numbers are elevated) without damaging already-compromised airway epithelium, it might complement rather than compete with ICS and biologics.

### 6.2 Pyroptosis in asthma: the glucocorticoid-resistant dimension

NLRP3 polymorphisms associate with childhood asthma susceptibility (125), and elevated NLRP3 with caspase-1/4/5 characterizes neutrophilic asthma sputum (126). NLRP3-dependent pyroptosis appears particularly prominent in glucocorticoid-resistant, severe, and neutrophilic phenotypes (127)—precisely the populations with the most limited therapeutic options. A mechanistic finding with cross-disease relevance concerns the distinctive relationship between inflammasome activation and cell fate in neutrophils. As detailed in Section 4.1.3, inflammasome-activated neutrophils do not follow a single deterministic trajectory but rather distribute across a spectrum of outcomes: non-lytic IL-1β/IL-18 secretion with preserved viability (driven by GSDMD-N trafficking to azurophilic granule and autophagosome membranes rather than the plasma membrane) (128, 129); GSDMD-dependent NET extrusion without obligate progression to full lysis; or classical pyroptotic membrane rupture. The balance between these fates depends on the intensity of inflammasome activation, the subcellular distribution of GSDMD-N, and local oxidative and metabolic context. This fate heterogeneity represents a cellular mechanism potentially common to both neutrophilic asthma and T2-low COPD: a substantial fraction of inflammasome-activated neutrophils persist as chronic sources of cytokines and extracellular traps rather than self-eliminating through lytic pyroptosis, thereby evading both death-dependent resolution and corticosteroid suppression.

## 7 Extracellular traps as a third death-associated inflammatory program converging on COPD pathology

### 7.1 Nomenclature and mechanisms: NETosis, METosis, and EETosis

Extracellular traps (ETs) are web-like structures composed of decondensed chromatin decorated with granule-derived antimicrobial proteins, released by activated innate immune cells into the extracellular space. Originally described in neutrophils by Brinkmann and colleagues as neutrophil extracellular traps (NETs) (130), the phenomenon has since been extended to macrophages (METosis) and eosinophils (EETosis) (131, 132), establishing ET formation as a broadly distributed innate defence program with direct relevance across multiple inflammatory cell lineages implicated in COPD.

Two mechanistically distinct modes of NET release have been characterised (133). Lytic (“suicidal”) NETosis proceeds over 2–4 hours through NADPH oxidase-dependent ROS, PAD4-mediated histone citrullination, chromatin decondensation, and plasma membrane rupture. GSDMD serves as an essential executioner: neutrophil elastase cleaves GSDMD to generate pore-forming N-terminal fragments, and GSDMD-deficient neutrophils show impaired lytic NET release (84, 134)—directly coupling NET execution to pyroptotic machinery. Non-lytic (“vital”) NETosis occurs on a faster timescale (minutes to ~1 hour) through nuclear budding and vesicular trafficking, preserving neutrophil viability (135). A related route involves mitochondrial DNA expulsion, generating traps enriched in oxidised mtDNA that potently activate cGAS-STING and TLR9 (136).

Macrophage extracellular traps (METs) and eosinophil extracellular traps (EETs) follow broadly analogous principles. EET formation in particular frequently involves mitochondrial DNA release and is especially relevant in T2-high inflammatory contexts, where IL-5-primed eosinophils release mtDNA traps under alarmin and allergen stimulation (137), providing a cellular counterpart to neutrophilic NET release in the T2-low endotype. Importantly, COPD-specific evidence now directly implicates METs in this disease: neutrophil elastase at clinically relevant concentrations induces MET formation in blood monocyte-derived macrophages from COPD patients, providing the first direct demonstration of an NE–MET axis operative in COPD (138). This finding is mechanistically pivotal because it reveals that neutrophil-derived proteases — themselves downstream products of NET formation — can reciprocally trigger programmed chromatin release from macrophages, establishing a NET–MET feed-forward loop that amplifies the extracellular trap burden in COPD airways. This loop is particularly relevant in the bacterially colonised, neutrophilic T2-low endotype, where sustained NE release from activated neutrophils could continuously recruit macrophages into MET-forming states, further magnifying DNA-mediated inflammatory amplification via the cGAS-STING/TLR9 sensing mechanisms described in Section 7.3.

### 7.2 Extracellular traps in COPD: evidence and triggers

Cigarette smoke is a potent and reproducible inducer of NET formation. Sputum and bronchoalveolar lavage fluid from COPD patients contain elevated concentrations of NET-associated markers—citrullinated histone H3 (CitH3), myeloperoxidase–DNA complexes, and neutrophil elastase–DNA complexes—with levels correlating with disease severity, exacerbation frequency, and airflow limitation (139, 140). NET burden is particularly increased during acute exacerbations and in bacterially colonised patients, mechanistically linking ETs to both T2-low inflammatory profiles and the microbiome dimension discussed in Section 4.3 (140).

A recent demonstration by Zhang et al. substantially advanced this picture (141). In chronic cigarette smoke–exposure murine models, NET-derived DNA was shown to accumulate progressively in airway tissue and to drive sustained inflammation through engagement of cytosolic and endosomal DNA-sensing machinery in airway epithelial cells and dendritic cells. Genetic or pharmacological disruption of NET formation—via PAD4 inhibition, DNase I administration, or mitochondrial ROS scavenging—attenuated airway inflammation, reduced lung function decline, and suppressed downstream type I interferon and NF-κB-dependent cytokine output. This work establishes NETs not merely as a consequence of inflammation but as an active driver of chronic airway pathology operating in parallel with, and mechanistically linked to, pyroptotic and ferroptotic programs.

In the COPD airway, multiple triggers converge to induce ET formation: direct cigarette smoke exposure and its reactive aldehydes (139); bacterial pathogens including Haemophilus influenzae and Moraxella catarrhalis (140); neutrophil-priming cytokines IL-8, TNF-α, and IL-33 (88) — with IL-33 additionally functioning as a direct driver of eosinophil EETosis through the ST2–IL-5–mtROS axis (91); and crystalline particulates and microbial DAMPs engaging TLR and inflammasome pathways (84). Eosinophil EETosis has additionally been documented in T2-high COPD and asthma subsets (137). Notably, the first COPD-specific demonstration of EET formation established that sputum samples from both stable and exacerbating COPD patients contain EETs, with EET burden correlating with disease severity (142). This positions ET formation as an inflammatory output of both endotypes — with neutrophilic NETs predominating in T2-low disease and eosinophilic EETs contributing in T2-high disease — and reinforces the clinical feasibility of EET-based biomarkers for endotype stratification proposed in Section 9.2.

Viral infection, a predominant trigger of COPD exacerbations, has recently been established as a potent driver of NET formation in this disease context. In rhinovirus-infected COPD patients and murine models, virus-induced NETosis was shown to propagate neutrophilic exacerbation through DNA-sensor–dependent cascades, and DNase I treatment attenuated both airway inflammation and lung function decline (143). This finding mechanistically links NET release to the viral triggers of COPD exacerbations and extends the microbiome–immune framework discussed in Section 4.3: not only bacterial colonisation but also viral infection drives disease progression by engaging NET-associated cell death programs, positioning extracellular trap biology at the interface of both stable-state pathology and acute exacerbation.

### 7.3 DNA sensing as an amplification node: cGAS-STING and TLR9

The pathological consequence of sustained ET release is not limited to direct tissue damage through trap-associated proteases and histones. Increasingly, the released DNA itself—particularly oxidised mitochondrial DNA and long-lived cytosolic double-stranded DNA—functions as a pattern recognition ligand engaging cytosolic and endosomal nucleic acid sensors that amplify inflammation throughout neighbouring tissue (141, 144).

The cGAS-STING pathway senses cytosolic double-stranded DNA, generating the second messenger 2′3′-cGAMP that activates STING and triggers TBK1–IRF3-dependent type I interferon production alongside NF-κB-driven inflammatory gene expression. In chronic cigarette smoke exposure, cGAS-STING is progressively activated in airway epithelium, alveolar macrophages, and dendritic cells; STING-deficient mice show attenuated emphysema development and reduced alveolar destruction (144, 145). Oxidised mtDNA released during ETosis is a particularly potent cGAS ligand because it resists cytosolic DNase degradation and forms stable higher-order structures (136).

TLR9, localised to endolysosomal compartments, senses unmethylated CpG motifs characteristic of bacterial and mitochondrial DNA. NET-derived DNA internalised by neighbouring cells engages TLR9 to drive MyD88-dependent NF-κB activation and IRF7-dependent type I interferon production (141, 146). TLR9 engagement in COPD airway epithelium and dendritic cells provides a complementary inflammatory amplification route operating alongside cGAS-STING, and Zhang et al. specifically demonstrated that both sensors were required for the full inflammatory consequence of NET-DNA accumulation in smoke-exposed airways (141).

### 7.4 Convergence with pyroptosis and ferroptosis: a death-inflammation triad

Extracellular traps occupy an integrative position within the convergence framework advanced in this review, intersecting pyroptotic and ferroptotic programs at four mechanistically distinct nodes.

First, at the execution level, GSDMD pore formation is required for lytic NETosis (84, 134), and inflammasome signaling itself directly triggers NET extrusion through GSDMD-dependent mechanisms (84, 85). This shared execution architecture means that inflammasome activation in colonised airways (Section 4.3) and neutrophils in general (Section 4.1.3) can yield non-lytic cytokine secretion, NET release, and/or pyroptotic lysis as alternative outcomes of a unified upstream signaling program. The therapeutic corollary is that GSDMD-directed interventions would be expected to attenuate all three programs simultaneously, providing a particularly attractive intervention node for the neutrophil-dominated T2-low endotype where non-lytic IL-1β secretion, NETosis, and pyroptosis likely coexist as parallel outputs of the same inflammasome activation events.

Second, at the upstream trigger level, ferroptotic lipid peroxidation generates mitochondrial dysfunction and ROS that prime both canonical NETosis and mitochondrial DNA release (136). The xCT/GSH/GPX4 axis disruption central to ferroptotic–pyroptotic coupling (Section 8.1) creates the same oxidative milieu permissive for extensive ET formation, providing a unified oxidative trigger across all three death-associated inflammatory programs.

**Figure 5:**
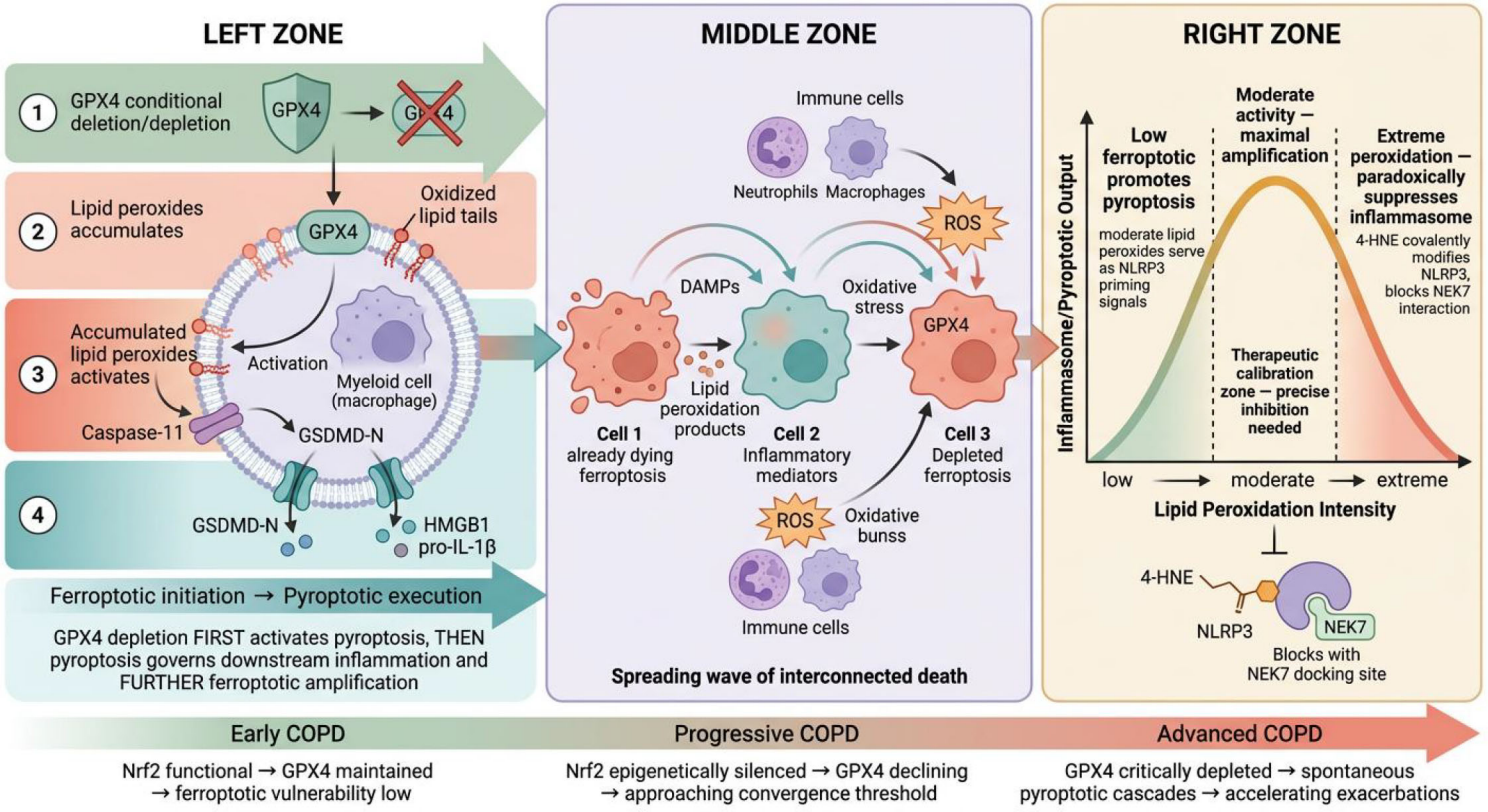
Mechanistic convergence of pyroptosis and ferroptosis: the GPX4 bridge, death propagation wave, and inverted U-shaped model. The left zone depicts the ferroptotic initiation-to-pyroptotic execution hierarchy: GPX4 depletion permits lipid peroxide accumulation that activates caspase-11–GSDMD pore formation, releasing HMGB1 and pro-IL-1β. The middle zone illustrates the intercellular death propagation wave, where ferroptotic DAMPs from Cell 1 trigger inflammatory amplification in Cell 2 and GPX4 depletion-driven ferroptosis in Cell 3, sustained by neutrophil and macrophage ROS bursts. The right zone presents the inverted U-shaped model: moderate lipid peroxidation maximally activates NLRP3 pyroptosis, while extreme peroxidation paradoxically suppresses inflammasome assembly through 4-HNE covalent modification blocking the NLRP3–NEK7 interaction. The bottom panel maps disease progression from early COPD (Nrf2 functional, GPX4 maintained) through progressive (Nrf2 epigenetically silenced) to advanced stages (GPX4 critically depleted, spontaneous pyroptotic cascades).

Third, at the amplification level, NET-derived oxidised mtDNA engages cGAS-STING and TLR9 to drive NF-κB and type I interferon signalling (144), which in turn upregulates NLRP3 inflammasome priming and suppresses Nrf2-dependent antioxidant defences—creating feed-forward loops that propagate pyroptotic and ferroptotic damage to neighbouring cells. DNA sensing therefore functions as a molecular bridge between ET-mediated damage and the inflammasome and ferroptotic programs detailed earlier, and reinforces the paracrine death-propagation wave described in the convergence model (**Figure 5**).

Fourth, at the therapeutic level, this convergence identifies several nodes amenable to simultaneous multi-pathway intervention: GSDMD inhibition (pyroptotic death and lytic NETosis); PAD4 inhibition (specifically NETosis) (141); DNase I-mediated NET clearance (removing the DNA substrate for downstream sensing) (141); cGAS-STING antagonism (blocking DNA-sensing amplification) (145); and mitochondrial-targeted antioxidants (suppressing mtDNA oxidation and release). In preclinical COPD models, DNase I administration and cGAS inhibition have shown additive effects when combined with antioxidant strategies (141, 145), supporting the principle that ET-targeting approaches complement rather than duplicate existing therapeutic avenues.

The integration of extracellular traps therefore reframes the destructive network in COPD as a triad rather than a dyad: pyroptosis, ferroptosis, and extracellular trap formation, each with distinct molecular execution machinery but sharing upstream oxidative triggers and downstream inflammatory consequences through DNA sensing and inflammasome priming. This triadic view preserves the endotype-specific mapping proposed earlier—NET-dominant inflammation in T2-low, bacterially colonised airways; EET contributions in T2-high, eosinophilic airways—while unifying all three programs within a single convergence logic that informs the therapeutic framework developed in Section 9.

## 8 Mechanistic convergence: where pyroptosis meets ferroptosis

### 8.1 GPX4 as a candidate molecular bridge between death programs: evidence and hypothesis

The proposition that GPX4 functions as a molecular bridge between ferroptosis and pyroptosis in COPD requires careful stratification by evidence strength rather than presentation as a settled mechanism. The strongest mechanistic evidence originates in non-COPD systems, where Kang and colleagues (147) provided the first causal demonstration that ferroptotic-type lipid peroxidation directly triggers pyroptotic execution. Using myeloid-specific Gpx4 deletion in mice, they showed that loss of GPX4 was sufficient—even without canonical inflammasome priming—to produce the full biochemical and morphological phenotype of pyroptosis, including GSDMD N-terminal cleavage, plasma membrane pore formation, propidium iodide uptake, HMGB1 release, and mature IL-1β secretion. Mechanistically, the oxidised phospholipids (particularly oxidised phosphatidylethanolamine species) that accumulated in the absence of GPX4 were shown to engage and activate caspase-11 directly, bypassing the requirement for sensor–adaptor inflammasome assembly. Rescue with vitamin E, ferrostatin-1, or GPX4 reconstitution established that lipid peroxide accumulation—not GPX4 loss per se—was the proximal trigger for pyroptotic execution, and the phenotypic consequence *in vivo* was a striking increase in polymicrobial sepsis lethality, reversible by pharmacological lipid radical trapping. Three principles emerged from this work that are central to the convergence framework: ferroptotic defence failure and pyroptotic execution are mechanistically coupled rather than parallel; GPX4 occupies the gatekeeping node whose loss is sufficient to initiate the cascade; and the coupling operates through lipid peroxide–caspase-11 physical engagement rather than through transcriptional priming. Complementary *in vitro* studies (18, 19) have subsequently shown that DAMPs released through ferroptosis-generated membrane defects propagate pyroptotic and necroptotic activation to neighbouring cells, adding a cell-to-cell amplification layer downstream of the primary GPX4-dependent event. We nevertheless emphasise that these foundational data derive from acute infection and sterile sepsis models in myeloid cells over hours-to-days timescales, and cannot alone establish that the same hierarchy operates in chronically injured airway epithelium.

The individual components of this hierarchy have now been independently documented in COPD models. Cigarette smoke induces ferroptosis in bronchial epithelial cells through iron accumulation and GPX4 suppression, with rescue by deferoxamine and ferrostatin-1 (106). Nrf2 undergoes progressive epigenetic silencing through CpG hypermethylation in COPD tissue, driving sustained GPX4 depletion (109). On the pyroptotic side, NLRP3 inflammasome components are elevated in COPD airways, correlate with disease severity and bacterial colonisation (73), and IL-1β concentrations in respiratory fluids parallel clinical deterioration (86, 87). The missing element—direct evidence that the two axes are mechanistically coupled in COPD-relevant tissue—has recently been supplied by Hou et al. (148). In cigarette smoke extract–exposed primary human bronchial epithelial cells and in chronic smoke-exposure murine models, they demonstrated that smoke inactivates the SLC7A11/xCT cystine–glutamate antiporter, producing cystine import failure, intracellular GSH depletion, and consequent GPX4 inactivation. The resulting accumulation of phospholipid hydroperoxides and 4-HNE was sufficient to drive GSDMD N-terminal cleavage, pyroptotic pore formation, and mature IL-1β release. Critically, ferrostatin-1 or pharmacological restoration of xCT function simultaneously suppressed both ferroptotic markers (MDA, 4-HNE, GPX4 degradation) and pyroptotic endpoints (cleaved GSDMD, IL-1β), whereas selective NLRP3 inhibition attenuated only the pyroptotic output—placing lipid peroxidation mechanistically upstream in airway tissue, exactly paralleling the hierarchy Kang et al. established in myeloid cells. Because this demonstration occurred in the epithelial compartment most directly exposed to inhaled smoke and used cigarette smoke extract rather than bacterial LPS or chemical GPX4 inhibitors as the upstream trigger, it provides the most COPD-relevant evidence to date that ferroptotic defence failure and pyroptotic execution are causally linked in the disease context. Taken together, Kang et al. (147) and Hou et al. (148) now form a mechanistically continuous evidence chain across acute infection and chronic inhalational injury, making the convergence model supported rather than purely extrapolative.

Several elements nevertheless remain to be clarified before clinical translation. Whether the Yang hierarchy operates predominantly through canonical caspase-1 activation (as their IL-1β data suggest) or also engages non-canonical caspase-11/4/5 signalling (as in the Kang sepsis paradigm) in different airway cell types has not been resolved, and parallel canonical priming through ROS/TXNIP-driven NLRP3 activation is almost certainly superimposed under smoke exposure. The temporal sequence of ferroptotic and pyroptotic marker emergence has not been characterised at single-cell resolution in human COPD tissue across GOLD stages; *in situ* co-localisation of GPX4, xCT, ACSL4, NLRP3, and cleaved GSDMD in bronchial biopsies—analogous to the NLRP3/PTGS2/ACSL4 analyses performed in coronary atherosclerosis (121)—has not been reported and would provide decisive histopathological confirmation. Whether interventional ferroptosis inhibition reduces pyroptotic output in alveolar macrophages, neutrophils and smooth muscle cells, and whether the Kang/Yang hierarchy contributes to exacerbations as opposed to stable-state progressive injury, also remain untested. On balance, we now describe GPX4 as an experimentally supported, emerging molecular bridge in COPD rather than an established one—a position strong enough to motivate dedicated therapeutic development but not yet sufficient to guide dose selection in human trials (**Figure 5**).

### 8.2 The lipid peroxidation paradox and its resolution

The most intellectually challenging aspect of this field is that lipid peroxidation exerts apparently contradictory effects on inflammasome regulation. The activating evidence is now substantial: Kang et al. (147) established in myeloid cells that GPX4 loss–driven phospholipid peroxide accumulation directly activates caspase-11 and GSDMD; Hou et al. (148) demonstrated in COPD airway epithelium that xCT/GSH/GPX4 axis disruption drives lipid peroxidation–dependent GSDMD cleavage and pyroptotic IL-1β release; octanal-induced lipid peroxidation enhances IL-1β output and promotes NLRP3-driven pathology (149); and CoQ10-mediated suppression of peroxidation prevents NLRP3 formation (150). Yet paradoxically, Hsu et al. (151) have shown that the peroxidation by-product 4-HNE can directly bind NLRP3 and prevent its interaction with NEK7, thereby inhibiting inflammasome activation, with intraperitoneal 4-HNE or RSL3 alleviating LPS-induced acute lung injury. These findings are only superficially contradictory. Once one recognises that “lipid peroxidation” is not a single parameter but a multi-dimensional phenomenon encompassing molecularly distinct species with different biological activities, the activating evidence from Kang and Yang and the suppressive evidence from Hsu can be understood as sampling different regions of the same underlying dose–response landscape.

#### 8.2.1 Defining the molecular parameter: what the inverted U describes

A critical clarification is required before the model can be properly evaluated: the inverted U-shaped relationship does not describe a single biochemical variable but rather reflects the net outcome of at least four distinct, non-interchangeable molecular parameters that shift in relative dominance as overall oxidative burden increases.

The first parameter is membrane-anchored phospholipid hydroperoxides (PLOOH), principally oxidised phosphatidylethanolamine (oxPE) species esterified into the plasma membrane. These are the direct enzymatic substrates of GPX4 and the first molecular species to accumulate when GPX4 activity is compromised. Kang et al. (147) demonstrated that these oxPE species physically engage the CARD domain of caspase-11, enabling oligomerisation and GSDMD cleavage independently of canonical inflammasome sensor-adaptor assembly. Hou et al. (148) showed that the same species accumulate in smoke-exposed bronchial epithelium upon xCT/GPX4 axis disruption and are sufficient to drive pyroptotic execution. Membrane PLOOH burden therefore constitutes the primary driver of the ascending limb of the inverted U: as PLOOH rises from physiological baseline, inflammasome/pyroptotic output increases proportionally through caspase-11 activation, GSDMD pore-mediated K^+^ efflux triggering secondary NLRP3 assembly, and DAMP release from permeabilised membranes.

The second parameter is free cytosolic 4-hydroxynonenal (4-HNE). 4-HNE is a secondary decomposition product generated when lipid hydroperoxide chains undergo β-scission; it is released from membranes into the cytosol, where its α, β-unsaturated carbonyl group confers high electrophilic reactivity toward protein cysteine, histidine, and lysine residues via Michael addition. Hsu et al. (151) demonstrated that exogenous 4-HNE covalently modifies specific cysteine residues on NLRP3 (particularly Cys-reactive sites within the NACHT domain), sterically blocking the NLRP3–NEK7 protein-protein interaction required for inflammasome oligomerisation. This covalent modification is irreversible under physiological conditions and directly suppresses inflammasome assembly independently of upstream signalling. Free cytosolic 4-HNE therefore constitutes the primary driver of the descending limb: once free 4-HNE accumulates to concentrations sufficient for significant NLRP3 modification (estimated from the Hsu data at >5–10 µM *in vitro*, though the *in vivo* threshold is unknown), inflammasome output paradoxically declines despite continued or increasing overall lipid peroxidation.

The third parameter is 4-HNE protein adduct burden. 4-HNE–protein adducts detected by immunohistochemistry (as in the Rahman et al. (118) COPD lung specimens) represent a cumulative, largely irreversible record of prior 4-HNE exposure. However, the adduct burden at any given time reflects the integral of past 4-HNE flux rather than the instantaneous concentration of reactive, free 4-HNE available for *de novo* NLRP3 modification. A cell with high 4-HNE adduct immunostaining may have low current free 4-HNE if aldehyde dehydrogenase and glutathione-S-transferase clearance pathways have subsequently reduced the free pool, or high free 4-HNE if clearance capacity has been overwhelmed. 4-HNE adduct burden is therefore an informative marker of cumulative oxidative history but does not directly predict whether a cell currently resides on the ascending or descending limb of the inverted U.

The fourth parameter comprises composite oxidative stress indices such as plasma or sputum MDA. MDA is a terminal decomposition product of PUFA peroxidation with a longer half-life than 4-HNE and broader substrate origin (it derives from PUFAs with ≥3 double bonds, whereas 4-HNE derives predominantly from ω-6 PUFAs). MDA measurements in clinical specimens reflect systemic or compartmental oxidative flux integrated across all cell types and membrane compartments and cannot resolve the subcellular distinction between membrane-anchored PLOOH (ascending limb driver) and free cytosolic aldehyde (descending limb driver) that determines inflammasome outcome. MDA and related composite indices (TBARS, F2-isoprostanes) are therefore useful as clinical stratification biomarkers for overall ferroptotic burden but insufficient as pharmacodynamic markers for calibrating therapy along the inverted U curve.

In summary, the ascending limb of the inverted U is primarily driven by parameter (i)—membrane PLOOH—while the descending limb is primarily driven by parameter (ii)—free cytosolic 4-HNE. Parameters (iii) and (iv) serve as clinically accessible but imprecise surrogates. This distinction is not merely semantic: it determines which molecular measurements are required to position an individual patient along the curve and which are insufficient (**Table 1**).

**Table 1:** Lipid peroxidation-associated parameters across COPD biological compartments: reported concentration ranges, measurement methods, and critical unknowns.

Parameter	Biological compartment	Reported range in COPD	Reported range in controls	Measurement method	Relevance to inverted U model	Critical unknowns
MDA (composite oxidative index, parameter iv)	Serum/Plasma	2.5–6.8 µmol/L (stable COPD); rises further during AECOPD	1.0–2.5 µmol/L	TBARS assay; HPLC-fluorescence	Low-resolution surrogate for overall ferroptotic burden; cannot distinguish ascending vs descending limb	Whether serum MDA reflects airway epithelial intracellular peroxidation levels; contribution of extrapulmonary sources; intra-patient variability on repeated sampling
	EBC	Elevated vs controls; absolute values variable due to dilution	Lower; poorly standardised	HPLC; colorimetric	Closer to airway compartment but highly variable	Standardisation of EBC collection; dilution factor correction; whether EBC MDA reflects epithelial or inflammatory cell origin
	Sputum	Elevated in COPD; quantitative data sparse	Limited data	TBARS; HPLC	Integrates epithelial, neutrophil, macrophage contributions	Cell-type-specific contribution unknown; mucus interference with assays
	Lung tissue	Not systematically quantified in COPD tissue homogenates	—	—	Would provide tissue-level reference	No published COPD tissue MDA concentration data by GOLD stage
Free 4-HNE (descending limb driver, parameter ii)	Serum/Plasma	Not systematically reported in COPD cohorts	0.3–0.7 µmol/L	HPLC-DNPH derivatisation; GC-MS	Would reflect systemic free aldehyde burden but not intracellular concentrations	COPD-specific serum free 4-HNE data essentially absent; relationship to intracellular free 4-HNE unknown
	BAL fluid	Not reported in COPD	—	—	Would approximate epithelial lining fluid concentration	No published data; dilution factor unknown; rapid clearance by albumin binding
	Intracellular (airway epithelium)	Not directly measured in COPD	Estimated 0.1–1 µmol/L in unstressed cells	Requires fluorescent probes or single-cell MS	This is the critical parameter for determining position on the inverted U curve	Completely unknown in COPD airway cells; whether the Hsu *in vitro* effective range (5–20 µmol/L) is achievable *in vivo* is untested
4-HNE protein adducts (cumulative marker, parameter iii)	Lung tissue (IHC)	Markedly increased in airway epithelium, alveolar epithelium, smooth muscle, endothelium; correlates inversely with FEV_1_	Present at low levels in smokers without airflow limitation	Immunohistochemistry (anti-4-HNE adduct Ab)	Cumulative oxidative history; maps cell compartments involved	Does not distinguish current from past oxidative burden; does not report free 4-HNE; no quantitative data linking adduct density to inflammasome suppression
	Serum	Elevated 4-HNE-modified proteins reported in COPD	Lower in controls	ELISA; Western blot	Systemic cumulative marker	Specificity for pulmonary versus hepatic/vascular sources
Membrane PLOOH (ascending limb driver, parameter i)	Lung tissue / airway epithelium	Not directly quantified in human COPD tissue	—	LC-MS/MS lipidomics (oxPE, oxPC species)	The primary driver of the ascending limb; the most mechanistically informative parameter	No published COPD data exist; all current evidence extrapolated from murine smoke models and *in vitro* CSE studies
	BAL fluid	Not reported	—	—	Phospholipids in BAL may partially reflect membrane shedding	No data; technical challenges of phospholipid oxidation artifact during sample processing
GPX4 protein	Lung tissue	Reduced protein expression in COPD vs controls	Higher expression	IHC; Western blot	Inverse proxy for PLOOH accumulation potential	Quantitative protein levels not standardised across studies; cell-type-specific expression in COPD biopsies not systematically mapped by GOLD stage
	Sputum cells	Not systematically reported	—	—	Would indicate GPX4 status in airway inflammatory cells	No published data
Iron homeostasis	Serum ferritin	Elevated in COPD	Lower	Immunoassay	Indirect indicator of iron loading favouring Fenton chemistry	Acute phase reactant—confounded by systemic inflammation
	BAL iron content	Reported elevated and correlating with severity	Lower	Atomic absorption; colorimetric	More specific to pulmonary compartment	Limited sample sizes; pre-analytical iron contamination risk
	Tissue TfR1 expression	Increased in airway epithelium	Lower expression	IHC	Indicates active iron uptake into epithelial cells	Functional iron pool (labile Fe²^+^) not measured; chelatable iron assays not performed in COPD biopsies

AECOPD, acute exacerbation of COPD; BAL, bronchoalveolar lavage; DNPH, dinitrophenylhydrazine; EBC, exhaled breath condensate; IHC, immunohistochemistry; LC-MS/MS, liquid chromatography–tandem mass spectrometry; oxPE, oxidised phosphatidylethanolamine; oxPC, oxidised phosphatidylcholine; PLOOH, phospholipid hydroperoxide; TBARS, thiobarbituric acid reactive substances; TfR1, transferrin receptor 1.

#### 8.2.2 Testable predictions, boundary conditions, and falsification criteria

We now state the inverted U-shaped model explicitly as a hypothesis with defined predictions and boundary conditions, rather than as a demonstrated mechanism.

The core hypothesis can be formulated as follows: in COPD airway cells, the relationship between total lipid peroxidation burden and NLRP3-dependent inflammasome/pyroptotic output follows a biphasic (inverted U-shaped) function, where the ascending phase is mechanistically driven by membrane PLOOH–caspase-11/caspase-1 engagement and the descending phase is driven by free 4-HNE covalent modification of NLRP3 preventing NEK7 docking.

This hypothesis generates the following testable predictions.

Prediction 1 (ascending limb validation): In primary human bronchial epithelial cells exposed to graded concentrations of cigarette smoke extract or titrated erastin/imidazole ketone erastin, increasing membrane PLOOH (quantified by LC-MS/MS lipidomics targeting oxPE species) should correlate positively with cleaved GSDMD, ASC speck formation, and mature IL-1β secretion up to a threshold concentration. This prediction can be tested within 12 months using existing air-liquid interface culture systems.

Prediction 2 (descending limb validation): Above the PLOOH threshold identified in Prediction 1, continued escalation of oxidative challenge should produce a measurable rise in free cytosolic 4-HNE (quantified by HPLC with dinitrophenylhydrazine derivatisation) concurrent with a decline in NLRP3–NEK7 co-immunoprecipitation signal and a plateau or decline in IL-1β output—despite continued increases in total lipid peroxidation markers (MDA, total 4-HNE adducts). Mass spectrometric identification of 4-HNE–NLRP3 adducts at the predicted cysteine sites would provide definitive molecular confirmation.

Prediction 3 (parameter dissociation): Manipulations that increase membrane PLOOH without proportionally increasing free 4-HNE (e.g., ACSL4 overexpression with aldehyde dehydrogenase inhibition) should produce maximal inflammasome activation without engaging the descending limb. Conversely, exogenous 4-HNE at the Hsu effective range (10–20 µM) without elevated membrane PLOOH should suppress inflammasome assembly without prior activation.

Prediction 4 (COPD stage mapping): Bronchial biopsies stratified by GOLD stage should show progressive increases in membrane PLOOH and 4-HNE adducts, with the free 4-HNE: PLOOH ratio increasing disproportionately in GOLD IV as aldehyde clearance capacity is exceeded—potentially correlating with blunted inflammasome responsiveness in end-stage disease.

Prediction 5 (therapeutic dose-response): In chronic smoke-exposure murine models, low-dose ferrostatin-1 should maximally attenuate pyroptotic markers, whereas high-dose ferrostatin-1 might paradoxically permit greater inflammasome activation from non-ferroptotic triggers normally suppressed by 4-HNE-mediated NLRP3 modification. This non-intuitive prediction provides the strongest falsification test.

Three boundary conditions delimit the model’s applicability. First, the descending limb is predicted to operate only on NLRP3-dependent and caspase-11-dependent inflammasome pathways, because the suppressive mechanism requires 4-HNE covalent modification of NLRP3-specific cysteine residues; AIM2, NLRC4, and PYRIN inflammasomes, which do not depend on NEK7 docking for assembly, should not exhibit descending-limb behaviour, and this provides a built-in specificity control. Second, the descending limb requires that free cytosolic 4-HNE reach concentrations sufficient for significant NLRP3 modification—a threshold that depends on cellular aldehyde dehydrogenase (principally ALDH2 and ALDH3A1) and glutathione-S-transferase (GSTA4) activity. Cell types with high aldehyde clearance capacity may never reach the descending limb even under severe oxidative stress, while those with low clearance capacity may enter it earlier. Third, the model describes a cell-autonomous effect; at the tissue level, paracrine amplification through DAMP release, immune cell recruitment, and ROS bursts from infiltrating neutrophils and macrophages creates a multicellular system whose apparent dose-response relationship may differ from the single-cell inverted U, potentially exhibiting a monotonic increase if immune-cell-derived PLOOH continuously re-primes inflammasomes in neighbouring cells faster than 4-HNE accumulates to suppressive levels.

Falsification criteria: The model would be substantially weakened if (a) graded xCT inhibition in primary human bronchial epithelial cells produces a monotonic (non-biphasic) relationship between PLOOH and IL-1β output across the full concentration range; (b) 4-HNE–NLRP3 adducts cannot be detected at the predicted cysteine residues in COPD lung tissue with high 4-HNE burden; or (c) NLRP3 constructs with cysteine-to-serine mutations at the Hsu-identified modification sites continue to show descending-limb behaviour, indicating an alternative suppressive mechanism.

Four axes distinguish the ascending and descending regions of this landscape beyond the molecular parameter identity discussed above. The first—lipid species identity—has been detailed in Section 8.2.1. The second axis is subcellular compartment: membrane-localised peroxidation generates propagating radical chains, K^+^ efflux and DAMP release that collectively favour inflammasome assembly (the Kang/Yang phenotype), whereas cytosolic accumulation of free reactive aldehydes provides direct molecular access to assembled inflammasome components for covalent modification (the Hsu phenotype). The balance between these compartments depends on ACSL4/LPCAT3-driven PUFA esterification versus aldehyde dehydrogenase clearance capacity. The third axis is concentration regime: the Kang and Yang data describe behaviour on the ascending limb—moderate, chronic, membrane-anchored peroxidation amplifying pyroptosis—whereas the Hsu data (151) describe the descending limb attained only at high exogenous free-aldehyde exposures. The Hou observations are particularly informative because they were obtained under chronic smoke exposure mimicking human COPD pathogenesis, suggesting that COPD airway tissue likely operates predominantly on the ascending limb during stable disease, with potential excursion to the descending limb only during severe exacerbations or end-stage disease when aldehyde clearance is overwhelmed. The fourth axis is temporal dynamics: acute oxidative bursts over hours (147), chronic sustained peroxidation over weeks-to-years (148), and supra-physiological aldehyde spikes may engage distinct downstream consequences—sublytic GSDMD activation with secretory IL-1β output, full pyroptotic lysis, or paradoxical termination of signalling through direct NLRP3 modification, respectively.

The therapeutic implication of ascending-limb operation for most COPD patients is that pharmacological ferroptotic inhibition—rather than maximisation of lipid peroxidation—should attenuate pyroptotic output. This is consistent with the rescue effects documented by Kang et al. (147) (ferrostatin-1 in sepsis) and Hou et al. (148) (ferrostatin-1 and xCT restoration in smoke-exposed epithelium). The descending-limb evidence nevertheless cautions that complete ablation of lipid peroxidation may not be required and could forfeit homeostatic signalling functions; the model predicts that partial ferroptotic inhibition calibrated to restore membrane PLOOH levels to a physiological window—while maintaining sufficient basal 4-HNE for constitutive NLRP3 tone modulation—may be therapeutically optimal. This prediction is directly testable through the graded-dose experimental designs outlined in Predictions 1–5 above. Until such data are available, the therapeutic implications of this model—particularly the contention that ferroptotic inhibition should be pharmacologically calibrated rather than maximised—should be regarded as hypothesis-generating predictions rather than dosing principles. The framework nevertheless offers a coherent synthesis of the Kang, Yang and Hsu literatures that would otherwise appear contradictory, and it makes specific predictions that can be falsified through the experiments outlined above (**Figure 5**).

## 9 Therapeutic integration: from pathway knowledge to clinical strategy

### 9.1 Dual-modality agents: mechanistic promise versus clinical reality

The convergence framework predicts that agents simultaneously affecting both death programs should show enhanced therapeutic efficacy. Several compounds demonstrate this property preclinically, though a critical appraisal of their clinical translatability in COPD reveals a substantial gap between mechanistic promise and deliverable therapy.

MCC950 (also known as CRID3 or CP-456, 773) suppresses caspase-1 and ASC activity to reduce IL-1β and IL-18 (152), and in sepsis models elevated GPX4 while reducing PTGS2 and 4-HNE (153), providing proof-of-concept for bidirectional targeting through a single molecule. However, the clinical development of MCC950 itself was discontinued following hepatotoxicity signals observed during phase II trials for rheumatoid arthritis in the late 1990s, and no MCC950 derivative has advanced to registered clinical trials in COPD. Second-generation NLRP3 inhibitors with improved pharmacokinetic and safety profiles — including dapansutrile (OLT1177, oral), inzomelid, somalix, and emlenoflast — have entered clinical development primarily for gout, cardiovascular, and neurological indications; to our knowledge, none has a registered interventional trial in COPD as of this writing. The translational gap between demonstrated mechanism and COPD-specific clinical evidence is therefore substantial, and extrapolation from non-respiratory indications must account for differences in target tissue accessibility (inhaled versus systemic delivery), chronic dosing requirements, and the risk of impairing host defence against respiratory pathogens — a particular concern given the bacterial colonisation characteristic of T2-low disease (Section 4.3).

Hydroxychloroquine inhibits NLRP3-mediated pyroptosis (154) and blocks erastin-induced ferroptosis (155), but its clinical applicability is constrained by cumulative retinal toxicity, QT prolongation, and cardiomyopathy risk—liabilities that are non-trivial in a COPD population with elevated cardiovascular risk. The concentrations required for *in vitro* inhibition of NLRP3 and ferroptosis typically exceed clinically tolerable exposures, and no randomised COPD trial has been reported.

Natural products (Schisandrin B, Nootkatone, epicatechin) suppress NLRP3-dependent pathology in preclinical models (156–158) but lack human pharmacokinetic characterisation, standardised formulations, and adequately powered clinical trials. We treat these as mechanistic probes rather than near-term clinical options.

Iron chelators and lipid radical trappers constitute the principal ferroptosis-directed agents in preclinical COPD literature. Deferoxamine, deferasirox, and deferiprone are clinically approved for iron overload disorders (thalassaemia, transfusion-dependent anaemias), providing a known human safety profile. However, repurposing for COPD faces specific obstacles: systemic iron chelation in non-overloaded patients risks functional iron deficiency with impaired erythropoiesis and cytochrome function; deferoxamine requires parenteral administration, limiting chronic outpatient use; deferasirox carries renal, hepatic, and gastrointestinal liabilities that have led to boxed warnings. The more mechanistically attractive approach — localised pulmonary delivery of iron chelation or lipid radical trapping — has not advanced beyond experimental formulation. Ferrostatin-1 and liproxstatin-1, the prototypical laboratory ferroptosis inhibitors, have not entered clinical development in any indication; their metabolic instability and lack of validated human pharmacokinetic data place them firmly at the preclinical stage.

What remains genuinely missing from the therapeutic landscape is deliberate pharmaceutical design based on convergence principles — agents engineered to simultaneously target GPX4–inflammasome coupling, lipid peroxidation-dependent caspase-11 activation, and DAMP-mediated intercellular death signal propagation, with pharmacokinetic and safety profiles appropriate for chronic respiratory disease. The inverted U-shaped model proposed in Section 8.2, if validated, would provide essential design parameters for such agents, particularly the principle that maximal ferroptotic inhibition may be neither necessary nor optimal.

### 9.2 Precision medicine: matching cell death-targeting therapy to inflammatory endotype

The COPD field’s success with biomarker-guided treatment allocation—principally using BEC to identify ICS responders, with >100 cells/µL suggesting potential benefit and >300 cells/µL indicating high likelihood of response (114, 159, 160)—provides a template for extending precision medicine to cell death-targeting interventions. It is important to note, however, that GOLD uses BEC as a predictive biomarker for treatment response rather than as a deterministic endotype classifier, and that clinical context—including exacerbation frequency, pneumonia history, and smoking status—remains integral to treatment decisions (160). The inflammatory profiles discussed in this review should therefore be understood as probabilistic guides to predominant pathobiology rather than as rigid categories that uniquely specify a single underlying mechanism.

We propose that a parallel biomarker strategy could incorporate circulating markers of inflammasome activation (IL-1β, IL-18, GSDMD fragments), lipid peroxidation products (MDA, 4-HNE), iron homeostasis parameters (ferritin, transferrin receptor), and GPX4 expression as a complement to, rather than a replacement for, existing clinical assessment frameworks. The clinical logic is straightforward: patients with neutrophilic, T2-low disease—who respond poorly to ICS and eosinophil-targeted biologics—may represent ideal candidates for pyroptosis-targeted therapies, given the established associations between neutrophilic inflammation, bacterial colonization, and non-canonical inflammasome activation (Section 4.3). Conversely, T2-high patients with elevated BEC might benefit from approaches addressing both alarmin-driven T2 inflammation and the ferroptotic oxidative damage that progressively silences Nrf2. We emphasise that these proposed therapeutic matches remain hypothetical and would require prospective validation in biomarker-stratified trials.

This reasoning gains support from the mixed results of anti-IL-5 approaches in COPD. Mepolizumab and benralizumab showed largely negative primary endpoints, though higher BEC subgroups demonstrated benefit (161, 162). One interpretation is that eosinophil-targeting alone is insufficient because it does not address the upstream pyroptotic and ferroptotic processes that generate the inflammatory environment recruiting eosinophils. The anti-alarmin therapies currently under investigation—tezepelumab (anti-TSLP) and itepekimab (anti-IL-33)—may prove more effective precisely because they target molecules closer to the epithelial injury response where pyroptosis-driven alarmin release originates (88, 163).

Importantly, the biomarker candidates proposed here are not theoretical constructs. As summarised in Section 5.4, MDA, 4-HNE, and GPX4 have each been measured in COPD patient cohorts and show biologically coherent associations with FEV1 and disease stage. Combining these ferroptosis-associated markers with established pyroptotic readouts (IL-1β, IL-18) and with BEC would yield a multi-dimensional stratification tool rather than a single-axis categorisation. A plausible near-term step would be to nest such a biomarker panel within ongoing trials of anti-alarmin biologics or NLRP3-directed small molecules, so that endotype-specific responses can be mapped against underlying death-program activity. Whether the inverted U-shaped relationship between lipid peroxidation and inflammasome activity (Section 8.2) translates into a measurable MDA/4-HNE threshold guiding dose selection is, in our view, one of the most actionable questions raised by the convergence framework.

### 9.3 Biologics and the cell death perspective: evidence-graded interpretation

Dupilumab (anti-IL-4Rα monoclonal antibody). Regulatory status: FDA-approved 27 September 2024 and EMA-approved 2025 as add-on maintenance therapy for adults with inadequately controlled COPD and an eosinophilic phenotype (BEC ≥300 cells/µL). Pivotal evidence: BOREAS (NCT03930732, phase 3, met primary endpoint) and NOTUS (NCT04456673, phase 3, met primary endpoint) demonstrated annualised moderate-or-severe exacerbation reductions of 30% (rate ratio 0.70; 95% CI 0.58–0.86) and 34% (rate ratio 0.66; 95% CI 0.54–0.82), respectively, with FEV_1_ improvements of 83 mL and 68 mL at week 12 (164, 165). Evidence level for cell-death mechanism: Speculative; no study has directly demonstrated dupilumab modulation of pyroptotic or ferroptotic markers in COPD patients. The cell-death intersection hypothesis is generative and warrants direct testing in biomarker sub-studies.

Tezepelumab (anti-TSLP monoclonal antibody). Regulatory status: FDA/EMA-approved for severe asthma; not approved for COPD. COPD evidence: COURSE (NCT04039113, phase 2a) did not meet its primary exacerbation endpoint in the overall moderate-to-very-severe COPD population, though numerical benefit was observed in subgroups with higher BEC (163). No phase 3 COPD programme registered as of April 2026. Evidence level: Phase 2a, negative primary endpoint.

Itepekimab (anti-IL-33 monoclonal antibody). Regulatory status: Investigational; not approved for any indication. COPD evidence: Phase 2a trial (NCT03546907) reported 42% exacerbation reduction in former smokers but not current smokers (166). Confirmatory phase 3 trials AERIFY-1 (NCT04701983) and AERIFY-2 (NCT04751487) are ongoing; topline results anticipated 2025–2026. Evidence level: Phase 2a subgroup signal awaiting phase 3 confirmation.

Tozorakimab (anti-IL-33 monoclonal antibody, binds both IL-33^red^ and IL-33^ox^). Regulatory status: Investigational. COPD evidence: Phase 3 TILIA programme ongoing (NCT05166889, NCT05158387). Evidence level: Phase 3 in progress; no efficacy readout available.

### 9.4 Ensifentrine: approved therapy, uncharacterised cell death effects

Regulatory status: FDA-approved 26 June 2024 as nebulised maintenance therapy for adults with COPD. Pivotal evidence: ENHANCE-1 (NCT04535986) and ENHANCE-2 (NCT04542057), phase 3, both met primary endpoint: FEV_1_ improvements of 87 mL and 94 mL; exacerbation rate reductions of 36% and 43% (167). Evidence level for cell-death mechanism: Inferential only; the ENHANCE programme did not include inflammasome or lipid-peroxidation biomarkers, and no preclinical or clinical study has assessed ensifentrine’s effect on pyroptotic or ferroptotic endpoints in COPD. The cAMP–NLRP3 mechanism remains a hypothesis warranting dedicated biomarker sub-studies.

**Figure 6:**
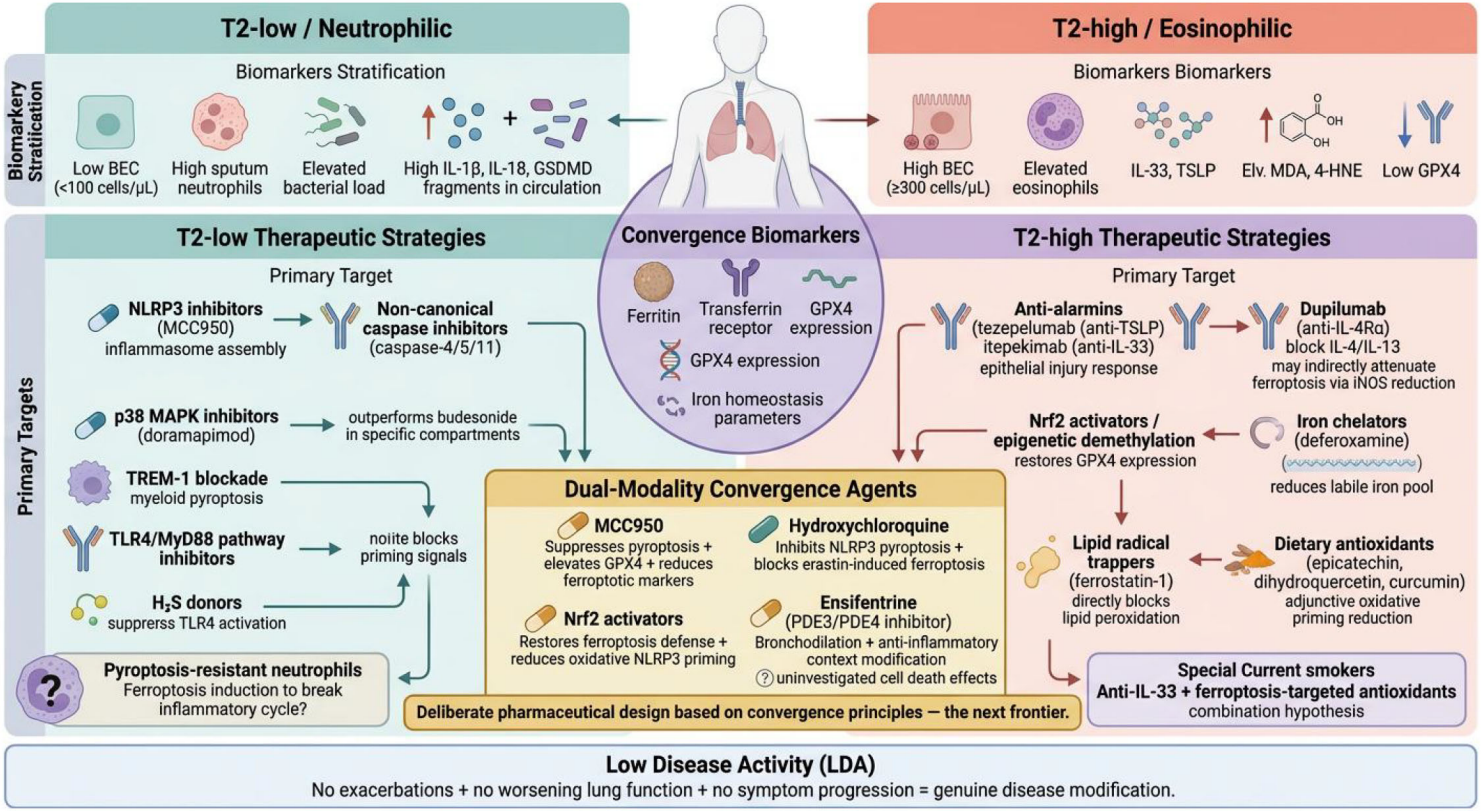
Therapeutic targeting of pyroptosis–ferroptosis crosstalk in COPD: a precision medicine framework. The therapeutic pipeline is organized by target pathway: pyroptosis-specific interventions (NLRP3 inhibitors, caspase-1 inhibitors, GSDMD-N blockers), ferroptosis-specific interventions (GPX4 activators, iron chelators, lipid peroxidation inhibitors), and dual-pathway strategies targeting shared nodes (Nrf2 activators, NF-κB modulators). Biomarker-guided patient stratification (center) matches endotype-specific death signatures to optimal therapeutic combinations. The timeline (bottom) indicates clinical development stages from preclinical through phase III trials. Dashed arrows indicate emerging combination strategies.

Compounds referenced as mechanistic probes (not approved for COPD). MCC950 (CRID3/CP-456, 773): clinical development discontinued in the 1990s due to hepatotoxicity; no derivative in registered COPD trials. Dapansutrile (OLT1177): phase 2 in gout/heart failure (NCT03534297); no registered COPD trials. Inzomelid, somalix, emlenoflast: phase 1–2 in non-respiratory indications. Ferrostatin-1, liproxstatin-1: preclinical only; no human pharmacokinetic data. Deferoxamine/deferasirox/deferiprone: approved for iron-overload disorders; no registered COPD trials. Hydroxychloroquine: approved for rheumatic/autoimmune indications; no randomised COPD trials for exacerbation prevention (**Figure 6**).

## 10 Synthesis and future directions

This review has argued that pyroptosis and ferroptosis constitute an integrated destructive network in COPD, connected through GPX4 at the molecular level, through alarmin-mediated inflammatory amplification at the cellular level, and through microbiome-immune interactions at the tissue level. Rather than representing independent pathways that coincidentally operate within diseased airways, they form a hierarchical system where ferroptotic lipid peroxide accumulation—driven by smoke-induced Nrf2 silencing—progressively triggers pyroptotic cascades that release inflammatory mediators and DAMPs, which in turn recruit immune cells whose oxidative burst further depletes GPX4 in neighboring cells.

### 10.1 Limitations

Several limitations of the current evidence base should be acknowledged. First, the proposed ferroptosis-to-pyroptosis hierarchy via GPX4–caspase-11 coupling has been demonstrated in sepsis models but has not yet been directly validated in COPD-specific cells or tissues; its applicability to the chronically inflamed airway therefore remains a testable hypothesis rather than an established mechanism. Second, the inverted U-shaped model relating lipid peroxidation intensity to inflammasome output is a conceptual synthesis drawn from disparate experimental systems, and no study has examined this dose–response relationship in COPD-relevant models. Third, our endotype-specific mapping—attributing T2-low disease primarily to non-canonical pyroptosis and T2-high disease to combined pyroptotic–ferroptotic pathology—is largely inferred from cross-sectional biomarker associations and *in vitro* pathway data; longitudinal studies directly linking cell death signatures to clinical trajectories within defined endotypes are lacking. Fourth, much of the therapeutic discussion relies on preclinical data, with most candidate agents (ferrostatin-1, MCC950, Nrf2 reactivators) lacking COPD-specific clinical trials, and the translational gap between mechanistic promise and clinical applicability remains substantial. Finally, the biomarker panel proposed for patient stratification (MDA, 4-HNE, GPX4, GSDMD fragments) has not been prospectively validated in combination, and its sensitivity and specificity for distinguishing death-program dominance in individual patients are unknown. These gaps collectively indicate that while the convergence framework offers a coherent and hypothesis-generating model, its clinical translation will require dedicated experimental and clinical validation.

### 10.2 Future directions

Translating this framework into therapeutic benefit requires addressing several unresolved questions. First, does the relative contribution of each death program shift across COPD stages? If pyroptosis predominates during early inflammatory phases while ferroptosis drives later progressive destruction, temporally sequenced interventions might outperform static regimens. The emerging emphasis on earlier intervention—before irreversible remodeling is established (168)—adds urgency to this question.

Second, the inverted U-shaped model relating lipid peroxidation intensity to inflammasome activation needs experimental validation. If confirmed, it would alter how ferroptosis inhibitors are dosed and timed.

Third, the heterogeneous fate of inflammasome-activated neutrophils—distributed across non-lytic cytokine secretion, GSDMD-dependent NET extrusion, and lytic pyroptosis depending on local context (Section 4.1.3)—represents a cellular mechanism potentially common to both T2-low COPD and severe neutrophilic asthma, in which a meaningful fraction of neutrophils persist as sustained inflammatory sources rather than self-eliminating. Whether inducing ferroptosis specifically in these persistently activated neutrophils could redirect them toward regulated elimination and thereby break the inflammatory cycle is an attractive but technically demanding hypothesis that would require demonstration of selective ferroptotic vulnerability in neutrophils relative to structural airway cells, as well as confirmation that ferroptotic induction does not simply convert non-lytic secretion into NET release or pyroptotic lysis—either of which could amplify rather than resolve airway inflammation.

Fourth, the concept of low disease activity (LDA) and disease stability—defined as no exacerbations alongside no worsening of lung function or symptoms (168)—could serve as an integrative endpoint for evaluating cell death-modifying therapies. If pyroptotic and ferroptotic inhibition achieves sustained disease activity reduction, this would represent genuine disease modification rather than symptomatic management.

Finally, the field would benefit from deliberate integration of cell death research with precision biomarker strategies and the expanding pharmacological armamentarium of biologics and inhaled agents. The convergence of these disciplines creates an unprecedented opportunity to reshape COPD therapeutics—but only if they advance as interconnected rather than siloed endeavors.

### 10.3 Priority experimental and clinical validation agenda

To move the convergence framework from conceptual model toward clinical application, we propose the following concrete research priorities. At the cellular level, conditional GPX4 knockdown in cigarette smoke extract-exposed primary human bronchial epithelial cells and alveolar macrophages should be assessed for caspase-11 activation, GSDMD cleavage, and IL-1β release, with rescue experiments using ferrostatin-1 to confirm directionality. Time-resolved single-cell imaging of ferroptotic and pyroptotic marker emergence in smoke-exposed air–liquid interface cultures would establish the temporal hierarchy between the two death programs. At the tissue level, multiplexed immunohistochemistry co-localising GPX4, ACSL4, NLRP3, and cleaved GSDMD in bronchial biopsies stratified by GOLD stage and inflammatory endotype would test whether convergence markers co-exist in the diseased human lung *in situ*. At the animal model level, chronic smoke-exposure studies comparing graded doses of ferrostatin-1 alone, NLRP3 inhibitors alone, and their combination—with readouts including emphysema index, BALF cytokines, and Nrf2 methylation status—would directly evaluate the therapeutic advantage of dual-targeting over single-pathway inhibition. At the clinical level, the most immediately actionable step would be nesting a cell death biomarker panel (MDA, 4-HNE, GPX4, ferritin, IL-1β, IL-18, GSDMD fragments) within ongoing phase III trials of anti-alarmin biologics (itepekimab, tezepelumab) and NLRP3-directed small molecules, enabling *post-hoc* stratification of treatment response by underlying death-program activity. A dedicated proof-of-concept trial combining inhaled ferrostatin-class agents with ICS in current smokers with documented Nrf2 hypermethylation, using HDAC2 activity and exacerbation rate as co-primary endpoints, would directly test the ferroptosis–corticosteroid resistance hypothesis proposed in Section 5.3. Finally, Mendelian randomisation analyses leveraging common variants in GPX4, NLRP3, and ACSL4 within large COPD biobanks (UK Biobank, COPDGene) could provide orthogonal evidence for causal relationships between cell death pathway activity and clinical outcomes without the confounding inherent in observational designs.
